# High yield production of the antifungal proteins PeAfpA and PdAfpB by vacuole targeting in a TMV‐based expression vector

**DOI:** 10.1111/pbi.70093

**Published:** 2025-05-03

**Authors:** Adrià Bugeda, Xiaoqing Shi, Laia Castillo, Jose F. Marcos, Paloma Manzanares, Juan José López‐Moya, María Coca

**Affiliations:** ^1^ Centre for Research in Agricultural Genomics (CRAG, CSIC‐IRTA‐UAB‐UB) Barcelona Spain; ^2^ Instituto de Agroquímica y Tecnología de Alimentos (IATA‐CSIC) Paterna Valencia Spain; ^3^ Consejo Superior de Investigaciones Científicas (CSIC) Barcelona Spain; ^4^ Present address: Horticulture and Landscape College Tianjin Agricultural University Tianjin China

**Keywords:** antifungal proteins, TMV viral vector, vacuole, apoplast, phytopathogenic fungi, crop protection

## Abstract

Antifungal proteins (AFPs) derived from filamentous fungi show great potential against economically significant fungi that cause plant diseases and consequently threat food safety and security. This study focuses on the *Penicillium expansum* PeAfpA and *Penicillium digitatum* PdAfpB proteins and their activity against several phytopathogens. The AFPs were synthesized through a highly productive tobacco mosaic virus‐based expression vector in the fast‐growing model plant *Nicotiana benthamiana*, combining signalling sequences for apoplastic and vacuolar compartmentalization to increase yields. Adding a vacuolar signalling peptide from a *Nicotiana sylvestris* chitinase at the C‐termini of the AFPs in combination with an apoplastic N‐terminal signalling peptide from *N. benthamiana* osmotin significantly enhanced AFP yields without altering functionality. Results showed an improvement of ninefold for PeAfpA and 3,5‐fold for PdAfpB compared to constructs with only the apoplastic N‐terminal signalling. Transmission electron microscopy and immunogold labelling confirmed the localization of AFPs in both the apoplast and the vacuole, highlighting its compatibility with vacuolar environments. *In vitro* and *in vivo* assessments against key pathogenic fungi, including *Magnaporthe oryzae*, *Botrytis cinerea* and *Fusarium proliferatum*, revealed that the activities of easily purified PeAfpA‐ and PdAfpB‐enriched plant extracts closely mirrored those of their purified fungal counterparts. This innovative approach represents a notable advance towards the application of AFPs as effective, safe and environmentally friendly ‘green biofungicides’ for safeguarding crop and postharvest produce and could also be applied to control other pathogenic fungi that threat human health.

## Introduction

Fungi are widespread eukaryotic organisms which pose a threat to various living entities, being able to infect both humans and plants. Fungal infections can range in severity from mild to life‐threatening, even surpassing death rates from well‐known parasitic organisms such as Malaria (Bongomin *et al*., 2017). Phytopathogenic fungi threaten food security and safety by damaging crops and contaminating food with mycotoxins (Bebber and Gurr, 2015). Limited availability of antifungal agents, coupled with issues like the development of resistance, toxicity, and undesired side effects (Perfect, 2016) hinders efforts against pathogenic fungi. Hence, there is a need for new antifungal molecules and treatments with unique mechanisms of action and properties.

Antifungal proteins (AFPs) from fungal origin are promising candidates for therapeutical applications in agriculture and medicine (Garrigues *et al*., 2017; Huber *et al*., 2018; López‐García *et al*., 2012; Meyer, 2008). AFPs are small, cationic, cysteine‐rich proteins (CRPs) that contain six to eight cysteines that form three or four disulphide bonds, conferring a highly compact structure. Their inherent stability makes them resistant to heat, proteases and extreme pH conditions (Batta *et al*., 2009; Campos‐Olivas *et al*., 1995; Garrigues *et al*., 2017; Hegedüs and Marx, 2013). AFPs exhibit potent and specific antifungal activity against key human and plant fungal pathogens, requiring low concentrations (Garrigues *et al*., 2017; Huber *et al*., 2018; Marx *et al*., 2008; Tóth *et al*., 2016; Vila *et al*., 2001; Virágh *et al*., 2014). Moreover, they are not toxic for plant or mammalian cells (Garrigues *et al*., 2018; Moreno *et al*., 2006; Szappanos *et al*., 2006).

Currently, there are around 50 described AFPs, including the founder AFP from *Aspergillus giganteus* (AgAFP) and PAF from *Penicillium chrysogenum*, and others more recently identified like *Penicillium expansum* PeAfpA and *Penicillium digitatum* PdAfpB (Garrigues *et al*., 2016, 2018; Lacadena *et al*., 1995; Meyer and Jung, 2018). PeAfpA (XM_016741490) and PdAfpB (XM_014676483) are highly potent AFPs with strong antifungal activities against key phytopathogenic and mycotoxin‐producing fungi (Garrigues *et al*., 2018; Martínez‐Culebras *et al*., 2021). PeAfpA confers protection to tomato plants against *Botrytis cinerea*, and against *P. digitatum* and *P. expansum* in orange and apple fruits, respectively (Gandía *et al*., 2020; Garrigues *et al*., 2018). PdAfpB controls *B. cinerea* in tomato plants (Shi *et al*., 2019) and inhibits *Penicillium roqueforti* growth in sliced bread (Valero Abad *et al*., 2023). Although similar in size and sequence, PeAfpA and PdAfpB differ in their recently characterized multifaceted mechanisms of action (Bugeda *et al*., 2020; Giner‐Llorca *et al*., 2023a; Ropero‐Pérez *et al*., 2023). In summary, both PeAfpA and PdAfpB proteins represent promising candidates for the development of new biofungicides.

Antifungal proteins have been produced biotechnologically in various organisms, including *Pichia pastoris* (Garrigues *et al*., 2017; López‐García *et al*., 2010) and efficiently in filamentous fungi using regulatory elements from the *P. chrysogenum paf* or the *P. expansum afpA* genes (Gandía *et al*., 2022; Garrigues *et al*., 2018; Huber *et al*., 2018; López‐García *et al*., 2010; Sonderegger *et al*., 2016; Tóth *et al*., 2018; Virágh *et al*., 2014). In addition, other efficient, sustainable and safe production systems are being explored, including plants. Plants offer advantages as expression platforms: they use sunlight, are virtually free from human pathogens, and are easier to scale than other organisms. Plant‐based systems are ideal for producing cysteine‐rich AFPs needing disulphide bonds and proper folding. Previous studies show that expressing AFPs in plants leads to the accumulation of active proteins and improved resistance against fungal pathogens (Coca *et al*., 2004; Girgi *et al*., 2006; Moreno *et al*., 2005; Oldach *et al*., 2001). Viral expression vectors are a common strategy for using plants as biotechnological platforms. Plant virus‐based biotechnological tools serve different purposes, including protein production (Cordero *et al*., 2017; Mardanova *et al*., 2017; Uranga *et al*., 2023). The parasitic lifestyle of plant viruses involves strategies to spread and to efficiently hijack plant protein production. Additionally, they also suppress gene silencing defences, enabling efficient production of foreign compounds (Csorba *et al*., 2015).

In this study, we report a strategy to enhance AFP production. Previously, we developed a tobacco mosaic virus (TMV) derived expression system for PdAfpB, achieving high yields by targeting the protein to the apoplast in *Nicotiana benthamiana* (Shi *et al*., 2019). This was done by adding the osmotin signalling peptide (AP24sp) from *N. benthamiana* (Melchers *et al*., 1993) at the N‐terminus of the protein, mimicking the maturation process by removing the pro‐peptide region of the fungal gene, and enabling external expression, avoiding detrimental effects on plant cells. Here, we adapted the system to produce PeAfpA in the apoplast as well and improved yields for both AFPs by adding a vacuolar sorting signalling peptide (VS) from *Nicotiana sylvestris* chitinase (Sticher *et al*., 1993) to the C‐terminal end of the AFP sequence. We showed that these modifications preserve the antifungal activity of plant‐expressed AFPs. Finally, we tested plant protein extracts enriched in PeAfpA and PdAfpB and showed that they protect tomato plants and fruits from *Botrytis* grey mould, rice plants from blast disease caused by *Magnaporthe oryzae* and rice seeds from *Fusarium proliferatum* infections.

## Results

### Constructs for TMV‐based expression of AFPs with compartmentalization signalling peptides

**Figure 1:**
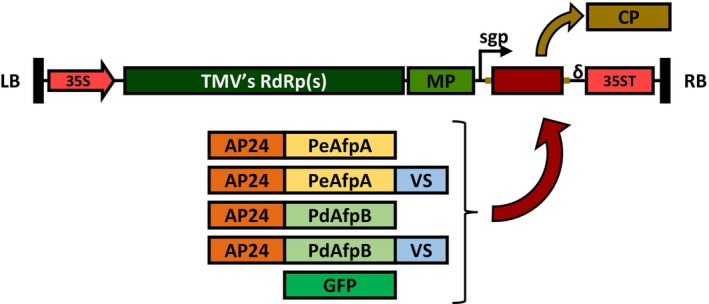
Schematic representation of the TMV‐based viral vector. The image shows the fragment of a binary plasmid for expression in plants comprised between the LB (Left border) and the RB (Right border), with the TMV's RdRp(s) (RNA‐dependant RNA polymerases), the MP (Movement Protein), CP's sgp (subgenomic promoter), everything under the control of 35S promoter and terminator sequences, preceded by a ribozyme sequence (∂). The image also shows how most part of the CP (Coat Protein) gene, with the original ATG mutated to AGA, is substituted by the protein of interest (PeAfpA, PdAfpB or GFP), fused to the apoplastic signalling peptide (AP24), and ending with or without the vacuolar sorting peptide (VS).

The strategy for the production and accumulation of PdAfpB in the apoplast was previously developed in our laboratory and described in (Shi *et al*., 2019). We constructed a TMV‐based vector in which the signalling peptide AP24sp was added at the N‐terminus of the protein of interest for its accumulation into the apoplast, contributing also to the maturation process by mimicking the fungal pro‐peptide removal. Here, a similar construct was made for PeAfpA production, and we also prepared new constructs for both PeAfpA and PdAfpB adopting a dual signalling strategy with the AP24sp at the N‐terminus serving for the maturation requirements, together with the incorporation of a vacuolar signalling peptide VS at the C‐terminus. An additional construct already available in the laboratory expressing the fluorescent protein GFP served to visually monitor the TMV‐based production process and as negative control for activity experiments (Figure 1).

**Figure 2:**
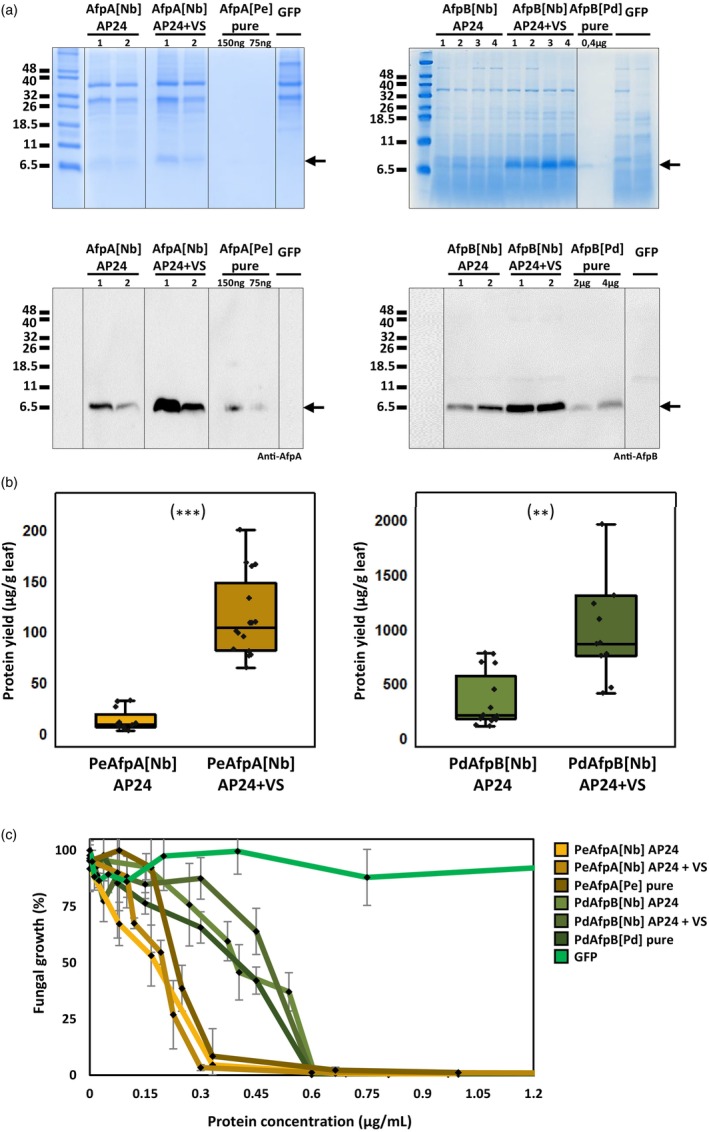
(a) Coomassie‐stained SDS gels and Western blots of plant extracts containing PeAfpA (left) and PdAfpB (right) produced in *Nicotiana benthamiana* ([Nb]) with the different constructs, or the purified proteins obtained from *Penicillium expansum* ([Pe]) or Penicillium digitatum ([Pd]). The last lane of each image belongs to extractions from *N. benthamiana* plants transformed with the GFP construct as a control. The amount of pure protein in the different PeAfpA and PdAfpB lanes is indicated. The lanes in gels and blots have been rearranged to improve presentation. (b) Box plots showing a comparison of the protein yields of different extractions of PeAfpA (left) and PdAfpB (right) corresponding to the two expression strategies, with each construct used indicated below the data. The dots correspond to a recompilation of minimum fifteen different extractions for each construct from different plants that were quantified by ELISA and densitometry. Asterisk denotes statistical difference between the two constructs (Student's *t*‐test ***P < 0.001; **P < 0.05). (c) Dose–response curves from antifungal assays in 96‐well microplates comparing the antifungal activity against *P. digitatum* of PeAfpA‐ (in yellow and brown colours, according to the legend) and PdAfpB‐enriched plant extracts (in dark green colours, according to the legend) compared with their purified counterparts produced in *P. expansum* or *P. digitatum* and GFP extracts as negative control (all identified by different colours). Growth was assessed after 72 h by measuring OD at 600 nm to calculate means and standard deviations, and values were obtained from at least two independent experiments, each with 4–6 replicates per concentration.

The cDNA used to produce the mature PeAfpA and PdAfpB proteins fused to the N‐terminal AP24sp were synthesized after codon usage adaptation for *N. benthamiana* and cloned, replacing most part of the viral coat protein (CP) into the binary plasmid containing the TMV vector. The remnants of CP with the initial ATG mutated to AGA were kept to preserve the regulatory regions in the genome of TMV and thus achieve a robust expression (Shi *et al*., 2019). These constructs are referred as AP24sp‐PeAfpA and AP24sp‐PdAfpB to indicate the incorporation of the N‐terminal signalling peptide, a modification intended to target proteins to the secretory pathway towards the extracellular space. The signal should be processed during trafficking (Melchers *et al*., 1993) and should be absent in the mature apoplastic protein. Constructs were agroinfiltrated into whole *N. benthamiana* plants by vacuum, and the produced proteins were later extracted with an acidic buffer suited for basic proteins such as AFPs. To assess production levels, plant protein extracts were analysed by Tricine‐SDS‐PAGE and Coomassie blue staining, and by Western blot using specific antibodies for each protein, comparing with similar extracts from plants infiltrated with the GFP control construct (Figure 2a). Protein bands with the expected molecular weights were observed in gels and specifically detected by antibodies in Western blot analysis. In Coomassie stained gels, the product of the AP24sp‐PeAfpA construct was often barely visible, but it was consistently detectable by Western blot.

We next examined the AFP production when potentially targeted also to vacuoles, testing the constructs AP24sp‐PeAfpA‐VS and AP24sp‐PdAfpB‐VS, engineered to combine the apoplastic secretory signal at the N‐terminus plus the vacuolar sorting signal (VS) of tobacco chitinases (Neuhaus *et al*., 1991) at the C‐terminus. The VS signal was intended to redirect proteins to the vacuole after their synthesis and to be processed upon its internalization to the compartment. Bands corresponding to both proteins with the expected molecular weights were observed at much higher intensities in samples of *N. benthamiana* plants agroinfiltrated and analysed as before (Figure 2a), indicating that PeAfpA and PdAfpB yields improved when the targeting signal to the vacuole was added.

### Protein production

AFPs from plant extracts were quantified initially by densitometry on Western blot membranes through comparison to known concentrations of pure PeAfpA or PdAfpB (Figure 2a). Values were also confirmed by ELISA with representative samples. Additionally, *in* vitro assays against *P. digitatum* (see below) were performed to confirm the activity and consistency with the concentrations reported in previous publications. Yields achieved from plants transformed with the same construct presented certain variability among different individual extractions, but the average was consistent after performing at least fifteen independent protein productions for each combination of protein and signalling peptides (Figure 2b).

Summarizing all available data, PeAfpA accumulated at an average of 12 ± 10 μg/g of fresh leaves when the plants where agroinfiltrated with the AP24sp‐PeAfpA construct. This average value was about 5 times lower than the yields reached by the same production system expressing the AFP from *A. giganteus* or the PdAfpB (Shi *et al*., 2019). These differences might suggest suboptimal expression or stability issues. However, the incorporation of the signal for compartmentalization into the vacuole in the AP24sp‐PeAfpA‐VS construct served to enhance the accumulation of the protein by more than 9 times, reaching an average value of 114 ± 40 μg/g of fresh leaves (Figure 2b).

The production of PdAfpB targeted to the apoplast yielded high amounts of protein, reaching 225 ± 37 μg/g of fresh leaves (Shi *et al*., 2019). Roughly comparable values were obtained in the present study with the apoplast‐targeted construct, with an average of 343 ± 253 μg/g. Those yields were indeed higher than most plant‐based systems used for AFPs production (Holaskova *et al*., 2015). Nonetheless, the vacuolar compartmentalization proved again to be an excellent strategy to produce AFPs, increasing the accumulation of PdAfpB by 3.5‐fold and reaching yields in the range of mg per g of tissue, with an average of 1182 ± 822 μg/g of fresh leaves (Figure 2b). It is noteworthy that the highest accumulation achieved with PdAfpB was roughly 10‐fold above the highest values obtained for PeAfpA (note the different axis scale in Figure 2b). At this stage, the observed yield variability across productions cannot be fully explained, as no clear correlations were identified between yield and common plant cultivation parameters. It is also unclear if instability issues might account for the observed high variability. Consequently, further research and optimization will be required to standardize the protocols.

### Antifungal activity of AFP‐containing plant extracts

Next, we assayed the *in vitr*o activity of the plant protein extracts resulting from the five constructs (two AFPs targeted to apoplast or apoplast and vacuole, plus the GFP control) against the filamentous fungus *P. digitatum*. As shown in Figure 2c, the growth inhibitory activity of the extracts containing PeAfpA or PdAfpB produced with the apoplastic or the apoplast+vacuolar constructs was equivalent to the purified proteins, reaching minimum inhibitory concentration (MIC) values of 0.3 μg/mL and 0.6 μg/mL, respectively, in contrast to GFP‐plant extracts which did not show any antifungal activity. These data demonstrate that the expression from constructs with the VS extension does not affect the antifungal activity of the proteins.

### Subcellular localization of the expressed PdAfpB

To explore the subcellular localization of the proteins produced with the viral vector, we decided to assess PdAfpB localization through transmission electron microscopy (TEM) and immunogold labelling. PdAfpB was chosen due to the higher yields reached and easier detection. For this purpose, we used agroinfiltrated leaves with the constructs AP24sp‐PdAfpB and AP24sp‐PdAfpB‐VS and compared them to leaves agroinfiltrated with the GFP construct and non‐agroinfiltrated leaves.

**Figure 3:**
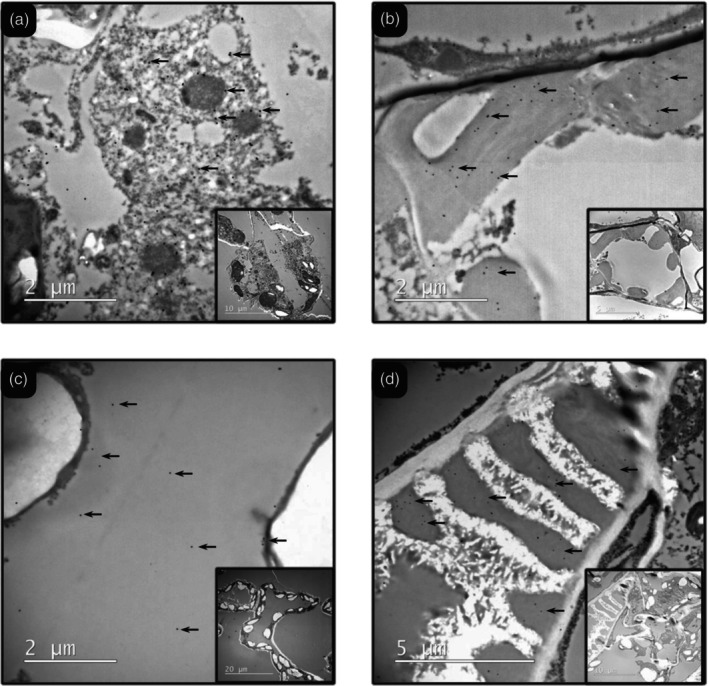
Representative transmission electron microscopy (TEM) images with immunogold labelling of PdAfpB. Black dots are the gold signalling (marked with black arrows). Images (a) and (b) are from leaves transformed with the AP24‐PdAfpB construct; (a) shows a fragment of a cell and (b) shows the tracheoles of the Xylem in the leaf's vessels. Images (c) and (d) are from leaves transformed with the AP24‐PdAfpB‐VS construct; (c) shows the vacuolar space of a plant cell and (d) shows the tracheoles of the Xylem of the leaves.

Observation of gold particles in tissues agroinfiltrated with AP24sp‐PdAfpB showed that PdAfpB accumulated mainly into the cytoplasm and in the xylem tracheoles. The presence of gold particles in the cytoplasm (Figure 3a) might correspond to recently produced proteins and probably still in transit towards the cell membrane. Interestingly, a remarkable amount of the gold signalling was also found in the xylem tracheoles (Figure 3b), lignified structures that help to maintain the integrity of the vessel.

On the other hand, PdAfpB produced with AP24sp‐PdAfpB‐VS was mainly detected in the vacuole (Figure 3c), and also some gold signalling was found again in the xylem tracheoles (Figure 3d). Most part of the signalling was found in the big, and apparently empty, central compartment that corresponded to the plant vacuole. The gold signals in the vacuole might seem more disperse compared with the density of dots in other structures like the xylem tracheoles, but it must be taken into consideration that the volume of the vacuole is much larger. Interestingly, almost no gold signals were detected in the cytoplasm of the leaves agroinfiltrated with the AP24sp‐PdAfpB‐VS construct, suggesting that the proteins labelled with the VS signalling peptide might be directed towards the vacuole at a much faster pace than those directed to the apoplast by the AP24sp. With regard to the signal observed in xylem vessels, caution is needed to evaluate this observation. Representative images of the control samples showed minimal detection of gold particles in the cytoplasm or vacuoles (Figure S2a). However, a small number of gold particles were detected in xylem tracheoles (Figure S2b), at a proportion approximately ten times lower than in the samples agroinfiltrated with the constructs expressing PdAfpB.

### Signalling peptide processing

Both the AP24 and the VS signalling peptides are expected to be processed upon targeting to the respective subcellular compartment destinations (Melchers *et al*., 1993; Sticher *et al*., 1993), and the similar electrophoretic mobility of plant‐produced AFPs and their fungal‐produced counterparts suggested that this was indeed the case. To confirm the expected processing of the signalling peptides, samples of the four constructs were analysed by mass spectrometry.

**Figure 4:**
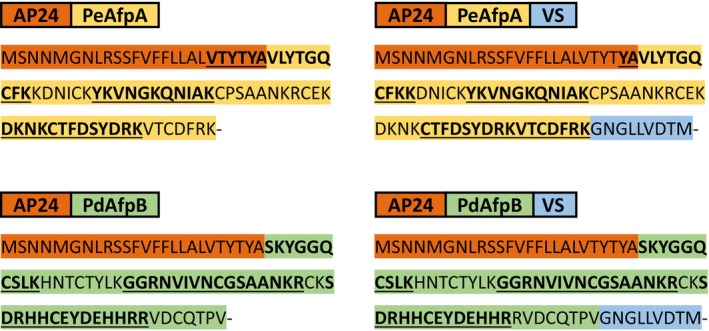
Peptides identified by mass spectrometry from PeAfpA and PdAfpB produced with the different constructs. The detected amino acids are underlined and marked in bold. Amino acids are highlighted in orange for the AP24 sequence, in blue for the VS sequence and in yellow and green for the PeAfpA and PdAfpB sequences. Further details can be found in Table S1.

Detected peptides by mass spectrometry are shown in Table S1 and summarized in Figure 4. Peptides covered around 60% and 70% of PeAfpA and PdAfpB sequences, respectively. The vacuolar signal VS was not detected in any of the proteins. Regarding AP24sp, it was not detected in PdAfpB constructs but part of its sequence (1 to 6 amino acids) was identified in a few of the peptides covering the N‐terminus of the mature PeAfpA, suggesting an incomplete processing.

### AFP protection of tomato plants and fruits against *B. cinerea* infection

**Figure 5:**
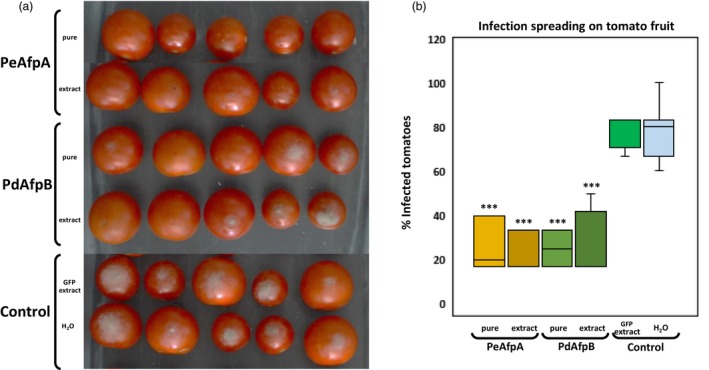
Effect of PeAfpA and PdAfpB on the infection of tomato fruits caused by *Botrytis cinerea*. (a) Representative image of treated tomatoes with purified or AFP‐enriched plant extracts at 7 dpi. GFP‐containing plant extracts or water were used as controls. (b) Box plot of the percentage of infected tomatoes. Five tomatoes per treatment in four different independent experiments were compared. Asterisks denote statistically significant differences in comparison to control values (Student's *t*‐test ***P < 0.001).

The effect of plant extracts containing the AFPs produced with both signalling peptides was tested on *B. cinerea* infection in tomatoes since both fungal proteins were described as active against this pathogen at concentrations as low as 2 μM (Garrigues *et al*., 2018). PdAfpB completely inhibited *B. cinerea* growth at concentrations as low as 2 μM, and PeAfpA at even lower concentrations, starting at 0.6 μM. Commercial tomato cherries were drop inoculated with *B. cinerea* conidia on needle‐prick wounds together with AP24sp‐PeAfpA‐VS‐ or AP24sp‐PdAfpB‐VS‐plant extracts, or together with the pure fungal proteins at the same concentrations (10 μM) and compared to control drops of conidia mixed with sterile water or GFP‐plant extracts as negative controls. We observed that negative control tomatoes were mostly infected at 7‐day post‐inoculation (dpi), with more than 80% of the wounds covered with grey mould (Figure 5a). However, tomatoes inoculated with drops containing AFPs, either as pure proteins or as plant extracts, were mostly non‐infected (Figure 5a). The quantification of infected tomatoes showed a statistically significant 50% reduction of grey mould incidence when treated with either pure proteins or plant‐enriched extracts (Figure 5b). These results demonstrated that both PeAfpA and PdAfpB are effective controlling *B. cinerea* infection in tomatoes and that also PeAfpA‐ and PdAfpB‐plant extracts can be used directly to control grey mould.

**Figure 6:**
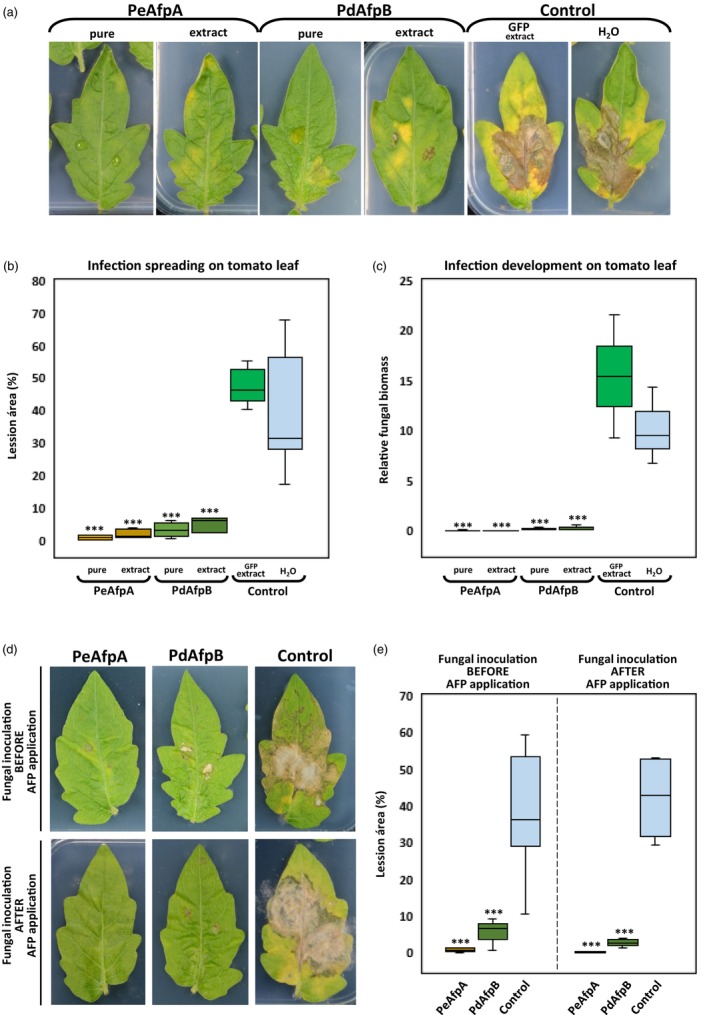
Tomato leaves protection against *Botrytis cinerea* infection by PeAfpA and PdAfpB. (a) Representative image of treated tomato leaves with purified or AFP‐enriched plant extracts. GFP‐containing plant extracts or water were used as controls. (b) Box plot of the percentage of leaf damage quantified by image analysis using the Fiji ImageJ2 package from at least three leaves per treatment from three independent assays. Asterisks denote statistically significant differences in comparison to control values (ANOVA and Tukey's HSD test ***P < 0.001). (c) Box plot of the fungal biomass quantified by qPCR. Asterisks denote statistically significant differences in comparison to control values (Student's *t*‐test ***P < 0.001 ). (d) Representative images of a tomato leaf protection assay. *B. cinerea* inoculation was done before (upper panel) or after (lower panel) protein treatment. The pictures show leaflets measuring 2.5–3 cm in width and 6–7 cm in length. (e) Box plot of the percentage of leaf damage from the experiment shown in the image (d) quantified by image analysis using the Fiji ImageJ2 package. Asterisks denote statistically significant differences in comparison to control values (ANOVA and Tukey's HSD test ***P < 0.001).

We also evaluated AP24sp‐PeAfpA‐VS‐ and AP24sp‐PdAfpB‐VS‐plant extracts in controlling *B. cinerea* infection in tomato leaves. In this case, we deposited the drops containing fungal conidia along with the antifungal proteins on detached tomato leaves without wounds. After 5 dpi, infection lesions were clearly visible in the control leaves, whereas no infection or only significantly smaller lesions were observed in the case of AFP‐treated leaves (Figure 6a). Through image analysis, we quantified a statistically significant decrease of 40% in the damaged area in PeAfpA‐ or PdAfpB‐treated leaves, either as pure proteins or as AFP‐containing plant extracts (Figure 6b). Area reduction correlated with fungal growth inhibition by AFPs as quantified by qPCR analysis of fungal DNA in inoculated leaves (Figure 6c). Furthermore, fungal biomass was barely detected on leaves treated with AFPs in contrast to control leaves. Moreover, we assessed PeAfpA and PdAfpB effectiveness when applied before and after fungal inoculation. We observed that both proteins were able to prevent *B. cinerea* infection when applied 24 h earlier than conidia inoculation, as well as to control fungal growth after 24 h infection (Figure 6d,e). Our results demonstrate that PeAfpA and PdAfpB, as well as PeAfpA‐ and PdAfpB‐plant extracts, confer protection against *B. cinerea* infection in tomato leaves.

### AFP protection of rice seeds against *F. proliferatum* infection

**Figure 7:**
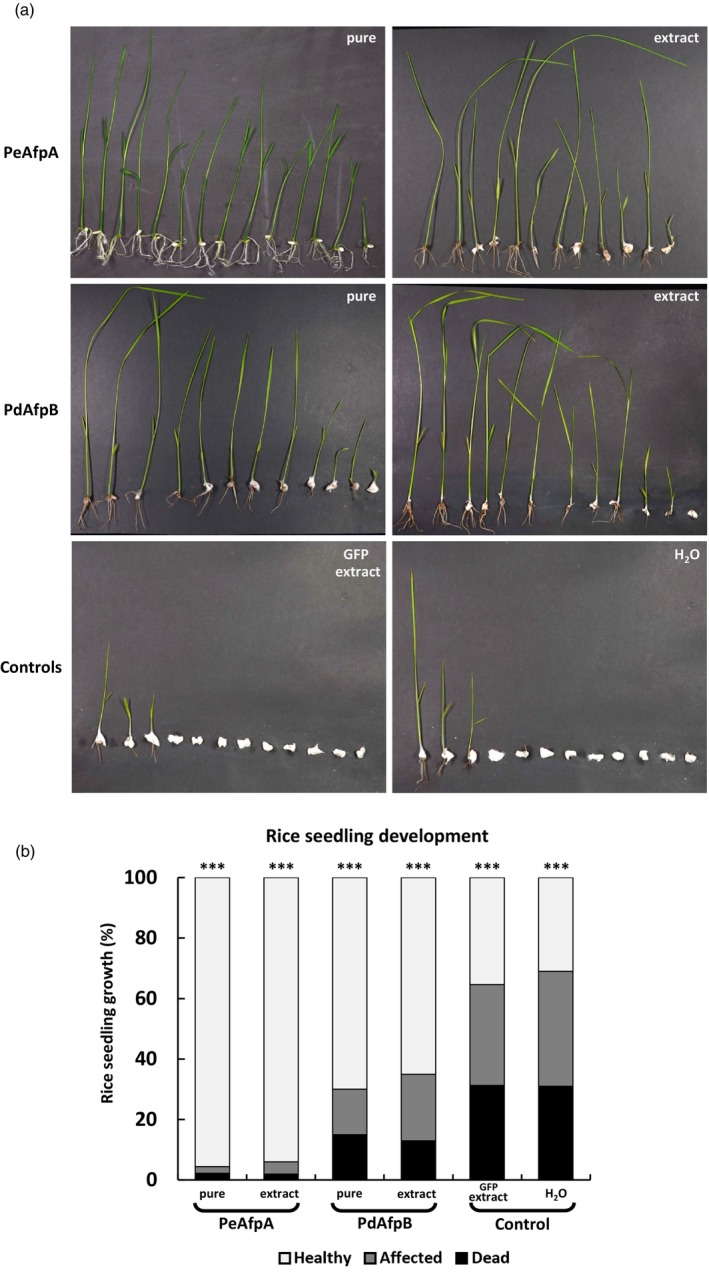
Protection of rice seeds against *Fusarium proliferatum* infection by PeAfpA and PdAfpB. (a) Representative image of a rice seed protection assay at 7 dpi. PeAfpA and PdAfpB were used as purified proteins or as AFP‐enriched plant extracts. GFP‐containing plant extracts and water were applied as controls. (b) Rice seedling growth at 7 dpi from all performed rice seed protection assays against *F. proliferatum* from at least 12 seeds per treatment from four different replicas. Asterisks denote statistically significant differences in comparison to control values (ANOVA and Tukey's HSD test ***P < 0.001).

PeAfpA is known to be particularly active against fungi of the *Fusarium* genus, showing MIC values as low as 1 μM, whereas PdAfpB requires concentrations in the range of 20 μM to completely inhibit their growth (Garrigues *et al*., 2017, 2018). *F. proliferatum* is associated with Bakanae rice disease, responsible for important crop losses and contamination of grains with mycotoxins (Wulff *et al*., 2010). Since this fungus inhibits rice seed germination, we evaluated the germination ability of drop‐inoculated seeds with conidia suspensions along with either pure PeAfpA or PdAfpB, or AFP‐containing plant extracts. Figure 7a showed that *F. proliferatum* was clearly affecting the germination and growth of seedlings in control conditions with around 70% of seeds completely dead or seriously affected in growth. By contrast, the presence of PeAfpA clearly inhibited fungal growth and as shown in Figure 7b, germination and growth were affected only in 5% of seeds. PdAfpB also improved seed germination and growth, although in a lower proportion than PeAfpA. Interestingly, the same protective effects were achieved by the PeAfpA‐ or PdAfpB‐enriched plant extracts compared to the respective pure proteins. These results suggest that PeAfpA and PdAfpB might be good alternatives for seed protection during storage and germination against *F. proliferatum* infection.

### AFP protection of rice leaves against *M. oryzae* infection

**Figure 8:**
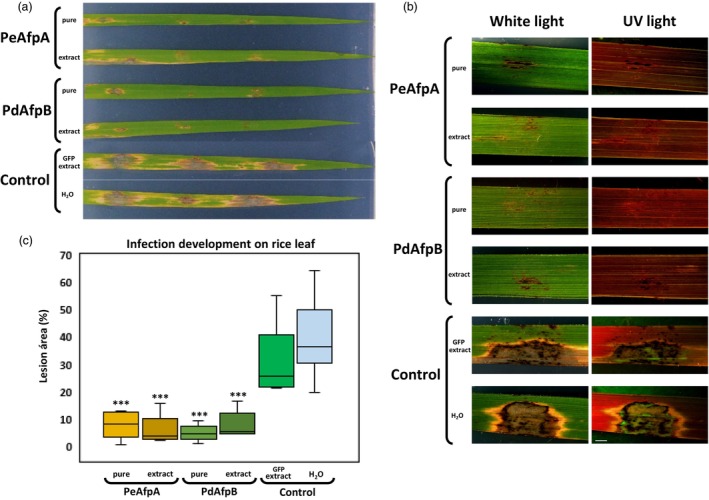
Protection of rice leaves against *Magnaporthe oryzae* by PeAfpA and PdAfpB. (a) Representative images of leaf symptoms at 7 dpi. PeAfpA and PdAfpB were applied as purified proteins or as AFP‐enriched extracts. GFP‐containing plant extracts and water were used as controls. (b) Representative images of lesions provoked by a GFP producer *M. oryzae* strain (*M. oryzae* Guy‐11 GFP), white light (left) or UV light (right). The width of rice leaves ranged from 0.5 to 0.7 cm. (c) Quantification of leaf lesion area for all performed rice leaf protection assays against *M. oryzae*. Lesion area was measured by image analysis using the Fiji ImageJ2 package. Three infection points on three leaves from three independent plants were analysed in at least three independent experiments. Asterisks denote statistically significant differences in comparison to control values (ANOVA and Tukey's HSD test ***P < 0.001).

Finally, we assessed the effectiveness of PeAfpA and PdAfpB against *M. oryzae* infection on rice leaves. This fungus is the causal agent of the blast disease, and it is highly susceptible to PeAfpA in *in vitro* assays with MIC values of 2 μM (Garrigues *et al*., 2018). AFP protection against the blast fungus was evaluated using a detached leaf infection assay. For the inoculation, drops of conidia suspensions were deposited along with AFPs on three different points of each leaf. At 6 dpi, the typical blast lesions with diamond shape and necrotic borders were observed in leaves inoculated with conidia in control conditions along with sterile water or GFP‐plant extracts (Figure 8a). No lesions or small necrotic lesions were detected when PeAfpA or PdAfpB were added along with the conidia, either as purified proteins or as plant extracts (Figure 8a). The quantification of the lesions by image analysis showed a statistically significant reduction on the lesion leaf area when AFPs were present (Figure 8c). A close inspection of lesions under UV light revealed the GFP‐labelled fungus growth on control lesions, which was not detected on the leaves with small necrotic lesions (Figure 8b). These data indicated that both PeAfpA or PdAfpB, either as purified proteins or as plant‐enriched extracts, could inhibit the growth and the infection process of *M. oryzae* on rice leaves, suggesting that they can also be used for the control of blast disease on rice plants.

## Discussion

In this study, we showed that using a TMV vector for transient expression and vacuolar targeting in *N. benthamiana* leaves is an efficient strategy for producing fungal AFPs at high yields. Our results indicate that the plant vacuole can be an excellent storage site for bioactive and stable compounds such as AFPs. Furthermore, we found that the incorporated signalling peptides were mostly processed during the production, with only minimal traces in some of the final analysed products. Additionally, we observed that the modifications for subcellular targeting did not affect the activity of the mature proteins, which were confirmed to store in the vacuole by TEM imaging. Plant extracts enriched with AFPs effectively protected crops, fruits and seeds from relevant fungal infections. The transient expression system is rapid, requiring just 7 days from plant agroinfiltration to protein extraction, and it does not require complex downstream purification steps. Therefore, it is fast, economic and scalable, since plants are inexpensive and easy to grow. Plants are also considered safe, being free from human pathogens. Altogether, this AFP production system can be an excellent starting point for developing control strategies in areas like crop protection, postharvest conservation or even clinical use, supported by the demonstrated *in vitro* activities (Garrigues *et al*., 2017, 2018). However, future applications must assess ecological impacts, such as effects on non‐target organisms and environmental persistence.

We also showed that the subcellular localization had a profound impact on protein yields. The mechanism of action of AFPs often relies on their ability for specific internalization in fungal cells, likely following an interaction with components of the fungal outer envelope, which could explain why they are usually not affecting other species (Bugeda *et al*., 2020; Giner‐Llorca *et al*., 2023b; Meyer and Jung, 2018). However, when produced ectopically inside the cells of a foreign organism, they can frequently result in toxicity and necrosis. Compartmentalization is known to help the accumulation of short antifungal peptides while avoiding their potentially toxic effects (Bundó *et al*., 2019; Coca *et al*., 2006; Montesinos *et al*., 2016). In a previous study, we found that PdAfpB provoked toxicity and failed to accumulate in *N. benthamiana* leaves when targeted to the endoplasmic reticulum, although it reached high yields when sorted to the extracellular space (Shi *et al*., 2019). Based on those results, we first approached the extracellular production of PeAfpA but observed a low accumulation accompanied by leaf wilting, suggesting that PeAfpA might have toxic effects in plants even when targeted outside the cells. The different outcomes for PeAfpA and PdAfpB might be determined by their different modes of action, which have been recently characterized for PdAfpB (Bugeda *et al*., 2020; Ropero‐Pérez *et al*., 2023) and PeAfpA (Giner‐Llorca *et al*., 2023a). The multifaceted mode of action of PdAfpB triggers a signalling cascade that leads to apoptosis‐like phenotypes. Both PdAfpB and PeAfpA are cell‐penetrating proteins for which protein O‐mannosylation is important for their interaction with the cell wall (CW). Regarding PeAfpA, we have described that the CW integrity (CWI) pathway is a key player in the killing mechanism against *Saccharomyces cerevisiae* (Giner‐Llorca *et al*., 2023a). PeAfpA is a highly active protein, surpassing PdAfpB, and its different and potent antifungal activity might be affecting plant cells during its synthesis and transit through the secretory pathway to the extracellular space, which might explain the low yields when targeted to the apoplast.

Fortunately, better yields were obtained when PeAfpA was targeted to the vacuole. The vacuole is by far the largest compartment in a plant cell; however, it is considered a hostile environment for protein accumulation due to its proteolytic activity. Nonetheless, PeAfpA and PdAfpB might be able to endure the lytic environment, perhaps as a direct consequence of their compact structures which have been shown to be quite resistant to the action of proteases (Garrigues *et al*., 2017). Our study proved the suitability of the vacuole as a reservoir compartment for the production of AFPs, improving protein yields by 9.2 times in the case of PeAfpA and by 3.5 times in the case of PdAfpB. By using TEM and immunogold labelling, we determined the subcellular localizations where the PdAfpB protein was ultimately stored, and indeed, it was found in the vacuole and also in the xylem tracheoles (Figure 3), suggesting that proteins secreted to the apoplastic space might end up accumulating in the xylem through internal hydraulic redistribution (Muller *et al*., 2014). However, the presence of a small number of gold particles in the tracheoles of control samples indicates nonspecific antibody binding. Detection in plant vacuoles has been rarely reported for the successful production of foreign proteins, mainly human proteins such as recombinant antibodies (Ocampo *et al*., 2016), collagen (Stein *et al*., 2009), complement factor C5a (Nausch *et al*., 2012) or transglutaminase (Marín Viegas *et al*., 2015). Our results add two AFPs to this list, demonstrating that vacuolar targeting can be an excellent strategy for increasing yields reaching levels of grams of protein per kilogram of fresh leaves.

To better characterize the new production system, mass spectrometry analyses were performed to determine if the signalling peptides were correctly processed. No presence of the VS signalling peptide was observed whatsoever on PeAfpA nor on PdAfpB, strongly suggesting that this peptide is completely eliminated during the transport process (Sticher *et al*., 1993). On the other hand, some amino acids from the AP24sp sequence were occasionally detected in a few peptides of both PeAfpA constructs, whereas no traces were observed in the PdAfpB constructs. The full‐size signalling peptide was never detected, and the small fragments observed did not match the predicted products of the trypsinization analytical treatment, suggesting incomplete processing. Even if the peptide was not fully processed, it could be later degraded partially by other means or remain in the protein, but, in any case, the PeAfpA‐containing extracts showed the expected activity. The *N. benthamiana* osmotin protein was the original source of the signalling peptide used in our synthetic constructs, and its 3D structure reveals a globular form from which the AP24sp protrudes (Min *et al*., 2004). The differences in signal peptide processing between the two proteins might be related to differences in their structure. The variable lengths of the remaining signalling peptide could result from unspecific degradation by plant proteases targeting fragments exposed from the globular and stable structure of the AFPs. However, the majority of the detected tryptic peptides from the N‐terminus of PeAfpA were not showing any rests of AP24sp, which ultimately suggests that, to a large extent, the final products from both PeAfpA and PdAfpB might be free from their signalling peptides, although further research might be required to fully understand this aspect of the peptide processing.

Our analysis showed that AFPs produced in plants using the new strategy were as active as their fungal‐purified counterparts. The finding that plant protein extracts were active in both *in vitro* and *in vivo* assays is very relevant, as it greatly simplifies downstream processing, which represent a significant part of the economic costs of other production methods (Wilken and Nikolov, 2012). Plant extracts highly enriched in active AFPs were obtained using an acidic buffer that selectively extracted basic proteins. Therefore, AFP physicochemical properties assisted the recovery of active products through an easy and efficient process that could reduce production costs significantly.

Regarding *in vivo* activity of the produced AFPs, we showed that application of plant extracts enriched either in PeAfpA or PdAfpB can confer protection against several relevant fungal pathogens in plants of agronomical interest, with rather potent activities similar to the observed with purified proteins. The quantities of AFPs required to completely prevent or significantly arrest fungal infections consistently were within the low micromolar range. This suggests treatments using spray applications of plant extracts could be economically viable.

The efficacy of peptide‐based fungicides by spray application has been reported recently for a legume nodule‐specific peptide used to control grey mould disease caused by *B. cinerea* in tomato and tobacco (Velivelli *et al*., 2020). We showed here that topical applications of the plant extracts enriched in PeAfpA and PdAfpB were highly effective in controlling *B. cinerea* infections in tomato plants and fruits, being PeAfpA more effective than PdAfpB. Further experiments would elucidate if these AFPs might also be active for the control of the grey mould disease provoked by this fungus in other important crop plants. *B. cinerea* is listed among the most important and economically relevant fungi (Dean *et al*., 2012). It is a broad‐spectrum phytopathogen that can infect numerous vegetable and fruit crops, including grapes, strawberries and tomatoes, causing serious losses during the pre‐harvest period and, more importantly, in post‐harvested fresh fruits, vegetables and flowers, with annual economic losses estimated between $10 to $ 100 billion worldwide (Weiberg *et al*., 2013). Given that botryoides represent around 10% of the fungicide market, the use of AFPs can have a significant economic impact as an alternative to complement the short list of available treatments for many vegetable and fruit crops.

We also showed that PeAfpA and PdAfpB were able to protect rice leaves against blast disease caused by *M. oryzae*, a hemibiotrophic fungus considered the most important fungal plant pathogen (Dean *et al*., 2012). This fungus provokes the severe and widely distributed rice blast disease, which is responsible for 10%–30% of grain losses (Skamnioti and Gurr, 2009). Since half of the world population relies on rice as a staple food, strategies to mitigate the devastating effects of the blast disease are very relevant. A previous report showed the efficacy of AgAFP on rice blast (Vila *et al*., 2001), suggesting that AFPs in general might be good alternatives to control blast disease in rice.

The fact that PeAfpA and PdAfpB were effective in different pathosystems and against fungi with different pathogenic strategies, suggests that they might be broadly used as natural fungicides in crop protection. Further studies will be needed to characterize the effectiveness of AFP treatments through foliar applications in greenhouse or field assays, their persistence over time and their effects on plant performance, including plant development and productivity.

Notably, PeAfpA‐ and PdAfpB‐enriched plant extracts also controlled *B. cinerea* infection in postharvest tomato fruits, supporting the use of AFP‐based fungicides for the management of postharvest decay on fresh fruits. Treatments with our plant extracts on fruits could become an economically feasible option since less amount of AFPs would be needed than for foliar applications. Previous reports demonstrated PeAfpA effectiveness controlling the green mould disease caused by *P. digitatum* in oranges (Garrigues *et al*., 2018) or the blue mould rot caused by *P. expansum* infection in Golden Delicious apples (Gandía *et al*., 2020). Given the fact that both PeAfpA and PdAfpB have potent fungicidal activities against a broad spectrum of fungal pathogens, we can anticipate that they could be widely applied for postharvest protection. Logically, the efficacy of each protein would need to be characterized for each pathosystem.

Finally, we also proved that plant extracts enriched in PeAfpA or PdAfpB were effective for controlling *F. proliferatum* infection in rice seeds. *Fusarium* species are relevant mycotoxigenic fungi that infect and colonize cereal crops and produce three of the most important mycotoxins commonly found in grains grown in America, Europe and Asia (Bennett and Klich, 2003). The increased germination rate of the seeds suggests that the AFPs might be protecting them from fungal infections that cause important losses through inhibition of germination. Although it needs to be experimentally determined, this protection could potentially be extended to other seeds and against other *Fusarium* spp., and perhaps even be applied as a seed surface coating. Additionally, *Fusarium* spp. might produce dangerous mycotoxins contaminating cereal grains used for human food and animal feed (Bennett and Klich, 2003; Summerell, 2019). Controlling *Fusarium* spp. along with other mycotoxigenic fungi such as *Aspergillus* spp. (Garrigues *et al*., 2017, 2018) could avoid mycotoxins entering the food chain.

While the system offers many positive outcomes like those described in previous paragraphs, it is important to recognize potential limitations and challenges. A first issue will be the scalability of *N. benthamiana* as a production system: expanding from laboratory to large‐scale production may involve economic and technical concerns, such as space requirements for plant cultivation and potential yield or quality variations due to environmental factors. Future research should focus on practical solutions, like optimizing plant growth conditions and engineering plants to reduce variability and enhance efficiency. Overcoming these challenges will be crucial for unlocking the full potential and sustainability of this technology.

In summary, our results at the laboratory scale showed a promising production strategy for PeAfpA and PdAfpB in plant biofactories and demonstrated their effectiveness in easy to obtain AFP‐enriched plant extracts, representing an important advance towards the future use of AFPs as environmentally friendly and effective ‘green fungicides’ for crop and postharvest protection against fungi.

## Experimental procedures

### Plasmid constructs

Antifungal proteins were produced in *N. benthamiana* leaves using the previously described TMV‐derived expression system (Shi *et al*., 2019). Three new constructs were prepared: first, the one for apoplastic PeAfpA expression, and later, the upgraded version for the expression of PeAfpA and PdAfpB in both the apoplast and the vacuole. The three constructs are represented together with the apoplastic PdAfpB and GFP producing constructs in Figure 1. Codon‐optimized cDNA encoding the mature PeAfpA protein (XP_016603682.1) extended in the N‐terminal with the secretory signal of tobacco AP24 protein (XP_009782398.1) was synthesized by Integrated DNA Technologies (Spain). For the apoplastic construct, the PeAfpA ORF was PCR amplified from the synthesized product using the primers CP:AP24_f and PeAfpA:CP_r (Table S1) and directly assembled through a Gibson reaction into the intermediate pMTMVi‐N plasmid as previously described (Shi *et al*., 2019). For the vacuolar construct, PeAfpA ORF was extended in the C‐terminal with the VS sequence through two consecutive rounds of PCR amplification, the first one using AP24_f and PeAfpAVS_r primers, and the second one using CP:AP24_f and VS:CP_r primers. The obtained DNA fragment was then assembled into the intermediate pMTMVi‐N plasmid. Equally, the AP24sp‐PdAfpB‐VS fragment was obtained through double PCR reactions, first using AP24_f and PdAfpBVS_r primers, and then, CP:AP24_f and VS:CP_r primers. Next, the TMV‐cDNA fragments in the intermediate plasmids were assembled into the pGTMV plasmid through a second Gibson reaction. All constructs were verified by sequencing.

### *N. benthamiana* leaf agroinfiltration

TMV recombinant clones in pGTMV plasmids were delivered into plants via agroinfiltration using *Agrobacterium tumefaciens* strain GV3101 carrying the pSoup helper plasmid (Hellens *et al*., 2000). Overnight cultures diluted at 0.1 OD_600_ in induction medium (10 mM MES, 10 mM MgCl_2_, 200 μM acetosyringone, 0.02% (v/v) Silwet L‐77) were incubated for 3 h at room temperature and used for vacuum infiltration of whole *N. benthamiana* plants. The plants were grown at the 4–5 leaf stage in the greenhouse at 24 °C with a 14 h light‐ 10 h dark photoperiod. Leaves of inoculated plants were harvested at 7 dpi for protein extraction.

### Protein extraction and analysis

Proteins were extracted from ECFs of fresh leaves by vacuum infiltration using phosphate saline buffer (PBS) supplemented with 0.02% (v/v) Silwet L‐77. Intracellular protein extracts were obtained from frozen leaves using acid buffer (84 mM citric acid, 30 mM Na_2_HPO_4_, 6 mM ascorbic acid, 0.1% (v/v) 2‐mercaptoethanol, pH 2.8). Extracts were clarified by centrifugation at 16 000 ***g*** for 15 min at 4 °C. Protein concentration was determined using the Bio‐Rad protein assay and bovine serum albumin (BSA) as standard.

Protein preparations were separated in tricine‐SDS‐PAGE (16.5%) and stained using Coomassie blue or transferred to a nitrocellulose membrane (Protran 0.2 μM) for immunodetection. PdAfpB was detected using antisera against *P. chrysogenum* PAFB kindly provided by Dr. F Marx (Huber *et al*., 2018), and PeAfpA with the antisera previously reported (Garrigues *et al*., 2018). Protein concentrations were determined by spectrophotometry at 280 nm or by comparing band intensities in complex protein extracts with known amounts of purified proteins using the Quantity Tools Image Lab™ Software (version 5.2.1) included in the ChemiDoc™ Touch Imaging System (Bio‐Rad, Spain).

### Antifungal assays

Growth inhibition assays against the *P. digitatum* CECT 20796 (PHI26) were performed in 96‐well microplates as previously described (Garrigues *et al*., 2017). Protein extracts and purified proteins were dialyzed against water and used at the indicated concentrations in antifungal assays. Growth was determined with measurements (OD 600 nm) every 24 h to calculate means and standard deviations. At least two independent experiments were performed, and the calculations included measurements after 72 h using 4–6 replicates per concentration.

### Enzyme‐linked Immunosorbent assay (ELISA)

ELISA was conducted following a modified protocol from Abcam™ on 96‐well microplates (Microplate 96 well High binding, Greiner Bio‐One™). The activated plate was incubated for 2 h with 100 μL of each sample at multiple dilutions to increase accuracy. Then, it was blocked with 5% non‐fat dry milk in T‐BST prior to incubation with anti‐PeAfpA or anti‐PdAfpB (1:10 000 and 1:2000 dilution, respectively) in PBS for 2 h at room temperature. Primary antibodies were detected with the secondary antibody Anti‐Rabbit IgG H&L (1:10 000 dilution) from goat coupled to the alkaline phosphatase (ab97048, Abcam™). After 2 h incubation at room temperature, plates were revealed using PNPP substrate (34 047, Thermo Scientific™) and evaluated using a plate spectrophotometer at 405 nm. Protein accumulation was estimated using a calibration curve of the purified version of each protein. Between each step, plates were washed 4 times with 200 μL T‐BST buffer per well.

### Sample preparation and immunogold staining for TEM

Plant tissue fragments were fixed with 4% paraformaldehyde and 0.1% glutaraldehyde in PB 0.1 M for 2 h at 4 °C and partially dehydrated in increasing concentrations of ethanol (30, 50 and 70%). Then, tissue fragments were embedded in LR White resin (Agar Scientific Ltd, Essex, UK) and polymerized at 50 °C for 24 h. Sections of 70 nm in thickness were obtained with a Leica EM UC6 ultramicrotome (Wetzlar, Germany) and collected with carbon film‐supported gold grids.

For immunogold procedures, all specimens were placed on carbon film‐supported gold grids. Sections were first rinsed with 1% Tween‐20 in PB 0.1 M for 10 min and then blocked with 5% BSA for 30 min. Next, specimens were incubated overnight with the primary antibody at adequate dilutions at 4 °C and washed prior being exposed to the secondary antibody EM goat anti‐rabbit IgG 20 nm (1:200) (BBI Solutions, Kent, UK) for 1 h at RT. Finally, sections were contrasted with 2% uranyl acetate for 30 s, and images were acquired in a Jeol JEM‐1400 (Jeol Ltd., Tokyo, Japan) at 80 kV.

### Mass spectrometry analysis

The analysis was performed in the proteomics service of the Scientific and Technological Centers at Universitat de Barcelona (CCiTUB). Protein samples were in‐gel digested after wash with 50 mM NH_4_HCO_3_ and acetonitrile (ACN), and reduction (DTT 20 mM) and alkylation (iodoacetamide 55 mM) treatments. Digestion was performed overnight with sequencing grade modified Trypsin (Promega), and the resulting peptide mixtures were extracted with 5% formic acid (FA) in 50% ACN and 100% ACN, dried in a SpeedVac and stored at −20 °C. The analysis was done in a nanoAcquity liquid chromatographer (Waters) coupled to an LTQ‐Orbitrap Velos (Thermo Scientific) mass spectrometer. Briefly, tryptic digests were resuspended in 1% FA solution and injected for chromatographic separation. Peptides were trapped on a Symmetry C18TM column (Waters) and separated using a C18 reverse phase capillary column (ACQUITY UPLC BEH column, Waters). Peptides were eluted with two different gradients with a 250 nL/min flow rate, and they were subjected to electrospray ionization in an emitter needle (Waters) with an applied voltage of 2000 V. Peptide masses (m/z 300–1600) were analysed in data dependent mode where a full Scan MS was acquired in the Orbitrap with a resolution of 60 000 FWHM at 400 m/z. Up to the 15th most abundant peptides (minimum intensity of 500 counts) were selected from each MS scan and then fragmented in the linear ion trap using CID (38% normalized collision energy) with helium as the collision gas. Generated raw data files were collected with Thermo Xcalibur (v.2.2) and used to search against a database of common laboratory contaminants with protein entries present in the UniProt public databases for *A. tumefaciens* complex, *Nicotiana* and Tobacco mosaic virus, together with the expected sequences for PdAfpB and PeAfpA variants, with Sequest HT search engine using Thermo Proteome Discoverer (v.1.4.1.14). The sensitivity of the search was improved using Percolator (semi‐supervised learning machine) to discriminate correct from incorrect peptide spectrum matches, assigning a q‐value defined as the minimal FDR at which the identification is deemed correct for every spectrum. The q‐values were estimated using the distribution of scores from the decoy database search. The search results were visualized in Thermo Proteome Discoverer (v.1.4.1.14) and filtered to only include proteins identified with at least two medium confidence peptides (FDR ≤ 0.05).

### Plant protection assays against fungal infection

For assays with tomato fruits, cherry tomatoes purchased in a local grocery store were first surface disinfected with a 1.5% (v/v) bleach solution for 5 min and profusely washed with abundant sterile water. Next, tomatoes were inoculated by placing 15 μL of *B. cinerea* conidia suspensions (5 × 10^5^ conidia/mL) onto needle‐prick wounds. To assess AFP protective effects, conidial suspensions were applied together with purified AFPs or plant protein extracts containing AFPs at 10 μM concentrations. Inoculated tomatoes were incubated at room temperature and high humidity in a box for 7 days to determine incidence percentages. We analysed 5 tomatoes per treatment in 4 independent experiments.

Tomato leaf protection assays against *B. cinerea* B05.10 infection were performed as previously described (Shi *et al*., 2019), applying the protein together with the fungus or applying the protein 24 h before the fungal inoculation. Infections were performed on detached tomato leaves of 3‐week‐old plants on 1% (w/v) agar in water containing 2 μg/mL kinetin. Leaves were locally infected at two points with conidial suspensions (10^6^ conidia/mL) by applying 20 μL drops containing 10 μM AFP solutions as purified proteins or as AFP‐enriched plant extracts. The progression of symptoms was followed visually. Lesion area was measured by image analysis using the Fiji ImageJ2 package. We analysed 2 infection points on 3 leaves from 3 independent plants in 3 independent experiments. Fungal biomass in inoculated leaves was determined at 3 dpi by qPCR using specific primers for BcCutA gene (Z69264) and normalized to tomato actin gene (U60480.1) (Table S2). DNA (15 ng per qPCR) was obtained using the Maxwell RSC plant DNA kit (Promega Biotech Ibérica, Spain). qPCR analyses were carried out in a LightCycler 480 System using SYBR green (Roche Diagnosis, Spain).

Rice seed protection assays against *F. proliferatum* were done on *Oryza sativa* var. Nipponbare seeds as previously described (Bundó *et al*., 2014; Gómez‐Ariza *et al*., 2007). Protection was assayed by inoculating seeds with 20 μL of spore suspensions (5 × 10^4^ conidia/mL) in the presence of purified AFPs or plant extracts containing AFPs at 10 μM concentrations. At 7 dpi, seedlings were categorized in 3 groups as: non‐germinated seeds, seedlings showing growth retardation and seedlings showing normal growth. We analysed 12 seeds per treatment in 4 independent experiments.

Blast disease protection was done using a detached leaf assay previously described (Coca *et al*., 2004). Leaves were collected from 4 leaves‐Nipponbare rice plants and drop‐inoculated with spore suspensions of *M. oryzae* Guy‐11 GFP (Sesma and Osbourn, 2004). Protection was tested by infecting with drops containing the fungal spores along with the antifungal proteins at 10 μM concentrations. The progression of symptoms was followed visually. Lesion area was measured by image analysis using the Fiji ImageJ2 package. Three infection points on 3 leaves from 3 independent plants were analysed in at least 3 independent experiments.

### Statistics

Statistical analysis was done using the R package (R‐3.6.1) to compute ANOVA and Tukey's HSD test at *P* values <0.001 and <0.05, or Student's *t*‐test at *P* values <0.05 and <0.01.

## Supporting information


**Figure S1** Sequences of pGTMV plasmid and AFP constructs.

**Figure S2** Control incubations for TEM experiments.

**Table S1** Mass spectrometry peptide analysis of expressed AFPs.

**Table S2** Primers used in this work.

## Data Availability

The data that support the findings of this study are available from the corresponding author upon reasonable request.
