# Supplementary material for: High yield production of the antifungal proteins PeAfpA and PdAfpB by vacuole targeting in a TMV‐based expression vector
Source: Plant Biotechnol J. 2025 May 3;24(1):313–27. doi: 10.1111/pbi.70093 (PMC12854906; doi:10.1111/pbi.70093)
Supplement: Supplementary file 1 — Figure S1 Sequences of pGTMV plasmid and AFP constructs. [file PBI-24-313-s001.pdf]

Supplementay Figure S1 Full sequence of the plasmid pGTMV, and sequence of the inserts corresponding to TMV-derived recombinant viruses.

pGTMV contains a full-length TMV infectious clone in a binary plasmid (GenBank accession number MK087763), and the sequences from the constructs of the TMV-based expression vector that substitute the CP for heterologous expression of the Gene of Interest (GoI).

>pGTMV (9669bp) – Binary plasmid containing the full sequence of the TMV virus

GCGGCCGCGATTCCATTGCCCAGCTATCTGTCACTTTATTGTGAAGATAGTGGAAAAGGAAGGTGGCTCC
TACAAATGCCATCATTGCGATAAAGGAAAGGCCATCGTTGAAGATGCCTCTGCCGACAGTGGTCCCAAAG
ATGGACCCCCACCCACGAGGAGCATCGTGGAAAAAGAAGACGTTCCAACCACGTCTTCAAAGCAAGTGGA
TTGATGTGATATCTCCACTGACGTAAGGGATGACGCACAATCCCACTATCCTTCGCAAGACCCTTCCTCT
ATATAAGGAAGTTCATTTCATTTGGAGAGGTATTTTTACAACAATTACCAACAACAACAAACAACAAACA
ACATTACAATTACTATTTACAATTACAATGGCATACACACAGACAGCTACCACATCAGCTTTGCTGGACA
CTGTCCGAGGAAACAACTCCTTGGTCAATGATCTAGCAAAGCGTCGTCTTTACGACACAGCGGTTGAAGA
GTTTAACGCTCGTGACCGCAGGCCCAAGGTGAACTTTTCAAAAGTAATAAGCGAGGAGCAGACGCTTATT
GCTACCCGGGCGTATCCAGAATTCCAAATTACATTTTATAACACGCAAAATGCCGTGCATTCGCTTGCAG
GTGGATTGCGATCTTTAGAACTGGAATATCTGATGATGCAAATTCCCTACGGATCATTGACTTATGACAT
AGGCGGGAATTTTGCATCGCATCTGTTCAAGGGACGAGCATATGTACACTGCTGCATGCCCAACCTGGAC
GTTCGAGACATCATGCGGCACGAAGGCCAGAAAGACAGTATTGAACTATACCTTTCTAGGCTAGAGAGAG
GGGGGAAAACAGTCCCCAACTTCCAAAAGGAAGCATTTGACAGATACGCAGAAATTCCTGAAGACGCTGT
CTGTCACAATACTTTCCAGACATGCGAACATCAGCCGATGCAGCAATCAGGCAGAGTGTATGCCATTGCG
CTACACAGCATATATGACATACCAGCCGATGAGTTCGGGGCGGCACTCTTGAGGAAAAATGTCCATACGT
GCTATGCCGCTTTCCACTTCTCCGAGAACCTGCTTCTTGAAGATTCATGCGTCAATTTGGACGAAATCAA
CGCGTGTTTTTCGCGCGATGGAGACAAGTTGACCTTTTCTTTTGCATCAGAGAGTACTCTTAATTACTGT
CATAGTTATTCTAATATTCTTAAGTATGTGTGCAAAACTTACTTCCCGGCCTCTAATAGAGAGGTTTACA
TGAAGGAGTTTTTAGTCACCAGAGTTAATACCTGGTTTTGTAAGTTTTCTAGAATAGATACTTTTCTTTT
GTACAAAGGTGTGGCCCATAAAAGTGTAGATAGTGAGCAGTTTTATACTGCAATGGAAGACGCATGGCAT
TACAAAAAGACTCTTGCAATGTGCAACAGCGAGAGAATCCTCCTTGAGGATTCATCATCAGTCAATTACT
GGTTTCCCAAAATGAGGGATATGGTCATCGTACCATTATTCGACATTTCTTTGGAGACTAGTAAGAGGAC
GCGCAAGGAAGTCTTAGTGTCCAAGGATTTCGTGTTTACAGTGCTTAACCACATTCGAACATACCAGGCG
AAAGCTCTTACATACGCAAATGTTTTGTCCTTCGTCGAATCGATTCGATCGAGGGTAATCATTAACGGTG
TGACAGCGAGGTCCGAATGGGATGTGGACAAATCTTTGTTACAATCCTTGTCCATGACGTTTTACCTGCA
TACTAAGCTTGCCGTTCTAAAGGATGACTTACTGATTAGCAAGTTTAGTCTCGGTTCGAAAACGGTGTGC
CAGCATGTGTGGGATGAGATTTCGCTGGCGTTTGGGAACGCATTTCCCTCCGTGAAAGAGAGGCTCTTGA
ACAGGAAACTTATCAGAGTGGCAGGCGACGCATTAGAGATCAGGGTGCCTGATCTATATGTGACCTTCCA
CGACAGATTAGTGACTGAGTACAAGGCCTCTGTGGACATGCCTGCGCTTGACATTAGGAAGAAGATGGAA
GAAACGGAAGTGATGTACAATGCACTTTCAGAATTATCGGTGTTAAGGGAGTCTGACAAATTCGATGTTG
ATGTTTTTTCCCAGATGTGCCAATCTTTGGAAGTTGACCCAATGACGGCAGCGAAGGTTATAGTCGCGGT
CATGAGCAATGAGAGCGGTCTGACTCTCACATTTGAACGACCTACTGAGGCGAATGTTGCGCTAGCTTTA
CAGGATCAAGAGAAGGCTTCAGAAGGTGCATTGGTAGTTACCTCAAGAGAAGTTGAAGAACCGTCCATGA
AGGGTTCGATGGCCAGAGGAGAGTTACAATTAGCTGGTCTTGCTGGAGATCATCCGGAGTCGTCCTATTC
TAAGAACGAGGAGATAGAGTCTTTAGAGCAGTTTCATATGGCGACGGCAGATTCGTTAATTCGTAAGCAG
ATGAGCTCGATTGTGTACACGGGTCCGATTAAAGTTCAGCAAATGAAAAACTTTATCGATAGCCTGGTAG
CATCACTATCTGCTGCGGTGTCGAATCTCGTCAAGATCCTCAAAGATACAGCTGCTATTGACCTTGAAAC
CCGTCAAAAGTTTGGAGTCTTGGATGTTGCATCTAGGAAGTGGTTAATCAAACCAACGGCCAAGAGTCAT
GCATGGGGTGTTGTTGAAACCCACGCGAGGAAGTATCATGTGGCGCTTTTGGAATATGATGAGCAGGGTG
TGGTGACATGCGATGATTGGAGAAGAGTAGCTGTTAGCTCTGAGTCTGTTGTTTATTCCGACATGGCGAA
ACTCAGAACTCTGCGCAGACTGCTTCGAAACGGAGAACCGCATGTCAGTAGCGCAAAGGTTGTTCTTGTG
GACGGAGTTCCGGGCTGTGGAAAAACCAAAGAAATTCTTTCCAGGGTTAATTTTGATGAAGATCTAATTT
TAGTACCTGGGAAGCAAGCCGCGGAAATGATCAGAAGACGTGCGAATTCCTCAGGGATTATTGTGGCCAC
GAAGGACAACGTTAAAACCGTTGATTCTTTCATGATGAATTTTGGGAAAAGCACACGCTGTCAGTTCAAG
AGGTTATTCATTGATGAAGGGTTGATGTTGCATACTGGTTGTGTTAATTTTCTTGTGGCGATGTCATTGT
GCGAAATTGCATATGTTTACGGAGACACACAGCAGATTCCATACATCAATAGAGTTTCAGGATTCCCGTA
CCCCGCCCATTTTGCCAAATTGGAAGTTGACGAGGTGGAGACACGCAGAACTACTCTCCGTTGTCCAGCC
GATGTCACACATTATCTGAACAGGAGATATGAGGGCTTTGTCATGAGCACTTCTTCGGTTAAAAAGTCTG

TTTCGCAGGAGATGGTCGGCGGAGCCGCCGTGATCAATCCGATCTCAAAACCCTTGCATGGCAAGATCCT
GACTTTTACCCAATCGGATAAAGAAGCTCTGCTTTCAAGAGGGTATTCAGATGTTCACACTGTGCATGAA
GTGCAAGGCGAGACATACTCTGATGTTTCACTAGTTAGGTTAACCCCTACACCGGTCTCCATCATTGCAG
GAGACAGCCCACATGTTTTGGTCGCATTGTCAAGGCACACCTGTTCGCTCAAGTACTACACTGTTGTTAT
GGATCCTTTAGTTAGTATCATTAGAGATCTAGAGAAACTTAGCTCGTACTTGTTAGATATGTATAAGGTC
GATGCAGGAACACAATAGCAATTACAGATTGACTCGGTGTTCAAAGGTTCCAATCTTTTTGTTGCAGCGC
CAAAGACTGGTGATATTTCTGATATGCAGTTTTACTATGATAAGTGTCTCCCAGGCAACAGCACCATGAT
GAATAATTTTGATGCTGTTACCATGAGGTTGACTGACATTTCATTGAATGTCAAAGATTGCATATTGGAT
ATGTCTAAGTCTGTTGCTGCGCCTAAGGATCAAATCAAACCACTAATACCTATGGTACGAACGGCGGCAG
AAATGCCACGCCAGACTGGACTATTGGAAAATTTAGTGGCGATGATTAAAAGAAACTTTAACGCACCCGA
GTTGTCTGGCATCATTGATATTGAAAATACTGCATCTTTGGTTGTAGATAAGTTTTTTGATAGTTATTTG
CTTAAAGAAAAAAGAAAACCAAATAAAAATGTTTCTTTGTTCAGTAGAGAGTCTCTCAATAGATGGTTAG
AAAAGCAGGAACAGGTAACAATAGGCCAGCTCGCAGATTTTGATTTTGTGGATTTGCCAGCAGTTGATCA
GTACAGACACATGATTAAAGCACAACCCAAACAAAAGTTGGACACTTCAATCCAAACGGAGTACCCGGCT
TTGCAGACGATTGTGTACCATTCAAAAAAGATCAATGCAATATTCGGCCCGTTGTTTAGTGAGCTTACTA
GGCAATTACTGGACAGTGTTGATTCGAGCAGATTTTTGTTTTTCACAAGAAAGACACCAGCGCAGATTGA
GGATTTCTTCGGAGATCTCGACAGTCATGTGCCGATGGATGTCTTGGAGCTGGATATATCAAAATACGAC
AAATCTCAGAATGAATTCCACTGTGCAGTAGAATACGAGATCTGGCGAAGATTGGGTTTTGAAGACTTCT
TGGGAGAAGTTTGGAAACAAGGGCATAGAAAGACCACCCTCAAGGATTATACCGCAGGTATAAAAACTTG
CATCTGGTATCAAAGAAAGAGCGGGGACGTCACGACGTTCATTGGAAACACTGTGATCATTGCTGCATGT
TTGGCCTCGATGCTTCCGATGGAGAAAATAATCAAAGGAGCCTTTTGCGGTGACGATAGTCTGCTGTACT
TTCCAAAGGGTTGTGAGTTTCCGGATGTGCAACACTCCGCAAATCTTATGTGGAATTTTGAAGCAAAACT
GTTTAAAAAACAGTATGGATACTTTTGCGGAAGATATGTAATACATCACGACAGAGGATGCATTGTGTAT
TACGATCCCCTAAAGTTGATCTCGAAACTTGGTGCTAAACACATCAAGGATTGGGAACACTTGGAGGAGT
TCAGAAGGTCTCTTTGTGATGTTGCTGTTTCGTTGAACAATTGTGCGTATTACACACAGTTGGACGACGC
TGTATGGGAGGTTCATAAGACCGCCCCTCCAGGTTCGTTTGTTTATAAAAGTCTGGTGAAGTATTTGTCT
GATAAAGTTCTTTTTAGAAGTTTGTTTATAGATGGCTCTAGTTGTTAAAGGAAAAGTGAATATCAATGAG
TTTATCGACCTGTCAAAAATGGAGAAGATCTTACCGTCGATGTTTACCCCTGTAAAGAGTGTTATGTGTT
CCAAAGTTGATAAAATAATGGTTCATGAGAATGAGTCATTGTCAGAGGTGAACCTTCTTAAAGGAGTTAA
GCTTATTGATAGTGGATACGTCTGTTTAGCCGGTTTGGTCGTCACGGGCGAGTGGAACTTGCCTGACAAT
TGCAGAGGAGGTGTGAGCGTGTGTCTGGTGGACAAAAGGATGGAAAGAGCCGACGAGGCCACTCTCGGAT
CTTACTACACAGCAGCTGCAAAGAAAAGATTTCAGTTCAAGGTCGTTCCCAATTATGCTATAACCACCCA
GGACGCGATGAAAAACGTCTGGCAAGTTTTAGTTAATATTAGAAATGTGAAGATGTCAGCGGGTTTCTGT
CCGCTTTCTCTGGAGTTTGTGTCGGTGTGTATTGTTTATAGAAATAATATAAAATTAGGTTTGAGAGAGA
AGATTACAAACGTGAGAGACGGAGGGCCCATGGAACTTACAGAAGAAGTCGTTGATGAGTTCATGGAAGA
TGTCCCTATGTCGATCAGGCTTGCAAAGTTTCGATCTCGAACCGGAAAAAAGAGTGATGTCCGCAAAGGG
AAAAATAGTAGTAGTGATCGGTCAGTGCCGAACAAGAACTATAGAAATGTTAAGGATTTTGGAGGAATGA
GTTTTAAAAAGAATAATTTAATCGATGATGATTCGGAGGCTACTGTCGCCGAATCGGATTCGTTTTAAAT
AGATCTTACAGTATCACTACTCCATCTCAGTTCGTGTTCTTGTCA [TCAGCGTGGGCCGACCCAATAGAG
TTAATTAATTTATGTACTAATGCCTTAGGAAATCAGTTTCAAACACAACAAGCTCGAACTGTCGTTCAAA
GACAATTCAGTGAGGTGTGGAAACCTTCACCACAAGTAACTGTTAGGTTCCCTGACAGTGACTTTAAGGT
GTACAGGTACAATGCGGTATTAGACCCGCTAGTCACAGCACTGTTAGGTGCATTCGACACTAGAAATAGA
ATAATAGAAGTTGAAAATCAGGCGAACCCCACGACTGCCGAGACGTTAGATGCTACTCGTAGAGTAGACG
ACGCAACGGTGGCCATAAGGAGCGCGATAAATAATTTAATAGTAGAATTGATCAGAGGAACCGGATCTTA
TAATCGGAGCTCTTTCGAGAGCTCTTCTGGTTTGGTTTGGACCTCT] GGTCCTGCAACTTGAGGTAGTCA
AGATGCATAATAAATAACGGATTGTGTCCGTAATCACACGTGGTGCGTACGATAACGCATAGTGTTTTTC
CCTCCACTTAAATCGAAGGGTTGTGTCTTGGATCGCGCGGGTCAAATGTATATGGTTCATATACATCCGC
AGGCACGTAATAAAGCGAGGGGTTCGAATCCCCCCGTTACCCCCGGTAGGGGCCCA**GGGTCGGCATGGCA**
TCTCCACCTCCTCGCGGTCCGACCTGGGCTACTTCGGTAGGCTAAGGGAGAAGCGCTGAAATCACCAGTC
TCTCTCTACAAATCTATCTCTCTCTATTTTCTCCATAAATAATGTGTGAGTAGTTTCCCGATAAGGGAAA
TTAGGGTTCTTATAGGGTTTCGCTCATGTGTTGAGCATATAAGAAACCCTTAGTATGTATTTGTATTTGT
AAAATACTTCTATCAATAAAATTTCTAATTCCTAAAACCAAAATCCAGGGGCCCTCGACGTTCCTTGACA
GGATATATTGGCGGGTAAACTAAGTCGCTGTATGTGTTTGTTTGAGATCCTCTAGGGCATGCAAGCTGAT
CTGGATCTCATGTGAGCAAAAGGCCAGCAAAAGGCCAGGAACCGTAAAAAGGCCGCGTTGCTGGCGTTTT
TCCATAGGCTCCGCCCCCCTGACGAGCATCACAAAAATCGACGCTCAAGTCAGAGGTGGCGAAACCCGAC
AGGACTATAAAGATACCAGGCGTTTCCCCCTGGAAGCTCCCTCGTGCGCTCTCCTGTTCCGACCCTGCCG
CTTACCGGATACCTGTCCGCCTTTCTCCCTTCGGGAAGCGTGGCGCTTTCTCATAGCTCACGCTGTAGGT

ATCTCAGTTCGGTGTAGGTCGTTCGCTCCAAGCTGGGCTGTGTGCACGAACCCCCCGTTCAGCCCGACCG
CTGCGCCTTATCCGGTAACTATCGTCTTGAGTCCAACCCGGTAAGACACGACTTATCGCCACTGGCAGCA
GCCACTGGTAACAGGATTAGCAGAGCGAGGTATGTAGGCGGTGCTACAGAGTTCTTGAAGTGGTGGCCTA
ACTACGGCTACACTAGAAGAACAGTATTTGGTATCTGCGCTCTGCTGAAGCCAGTTACCTTCGGAAGAAG
AGTTGGTAGCTCTTGATCCGGCAAACAAACCACCGCTGGTAGCGGTGGTTTTTTTGTTTGCAAGCAGCAG
ATTACGCGCAGAAAAAAAGGATCTCAAGAAGATCCTTTGATCTTTTCTACGGGGTCTGACGCTCAGTGGA
ACGAAAACTCACGTTAAGGGATTTTGGTCATGAGATTATCAAAAAGGATCTTCACCTAGATCCTTTTAAA
TTAAAAATGAAGTTTTAAATCAATCTAAAGTATATATGTGTAACATTGGTCTAGTGATTAGAAAAACTCA
TCGAGCATCAAATGAAACTGCAATTTATTCATATCAGGATTATCAATACCATATTTTTGAAAAAGCCGTT
TCTGTAATGAAGGAGAAAACTCACCGAGGCAGTTCCATAGGATGGCAAGATCCTGGTATCGGTCTGCGAT
TCCGACTCGTCCAACATCAATACAACCTATTAATTTCCCCTCGTCAAAAATAAGGTTATCAAGTGAGAAA
TCACCATGAGTGACGACTGAATCCGGTGAGAATGGCAAAAGTTTATGCATTTCTTTCCAGACTTGTTCAA
CAGGCCAGCCATTACGCTCGTCATCAAAATCACTCGCATCAACCAAACCGTTATTCATTCGTGATTGCGC
CTGAGCAAGACGAAATACGCGATCGCTGTTAAAAGGACAATTACAAACAGGAATCGAATGCAACCGGCGC
AGGAACACTGCCAGCGCATCAACAATATTTTCACCTGAATCAGGATATTCTTCTAATACCTGGAATGCTG
TTTTCCCTGGGATCGCAGTGGTGAGTAACCATGCATCATCAGGAGTACGGATAAAATGCTTGATGGTCGG
AAGAGGCATAAATTCCGTCAGCCAGTTTAGTCTGACCATCTCATCTGTAACAACATTGGCAACGCTACCT
TTGCCATGTTTCAGAAACAACTCTGGCGCATCGGGCTTCCCATACAATCGGTAGATTGTCGCACCTGATT
GCCCGACATTATCGCGAGCCCATTTATACCCATATAAATCAGCATCCATGTTGGAATTTAATCGCGGCCT
TGAGCAAGACGTTTCCCGTTGAATATGGCTCATAACACCCCTTGTATTACTGTTTATGTAAGCAGACAGT
TTTATTGTTCATGATGATATATTTTTATCTTGTGCAATGTAACATCAGAGATTTTGAGACACAACGTGGC
TTTGTTGAATAAATCGAACTTTTGCTGAGTTGAAGGATCAGATCACGCATCTTCCCGACAACGCAGACCG
TTCCGTGGCAAAGCAAAAGTTCAAAATCACCAACTGGTCCACCTACAACAAAGCTCTCATCAACCGTGGC
TCCCTCACTTTCTGGCTGGATGATGGGGCGATTCAGGCGATCCCATCCAACAGCCCGCCGTCGAGCGGG
CTTTTTTATCCCCGGAAGCCTGTGGATAGAGGGTAGTTATCCACGTGAAACCGCTAATGCCCCGCAAAGC
CTTGATTCACGGGGCTTTCCGGCCCGCTCCAAAAACTATCCACGTGAAATCGCTAATCAGGGTACGTGAA
ATCGCTAATCGGAGTACGTGAAATCGCTAATAAGGTCACGTGAAATCGCTAATCAAAAAGGCACGTGAGA
ACGCTAATAGCCCTTTCAGATCAACAGCTTGCAAACACCCCTCGCTCCGGCAAGTAGTTACAGCAAGTAG
TATGTTCAATTAGCTTTTCAATTATGAATATATATATCAATTATTGGTCGCCCTTGGCTTGTGGACAATG
CGCTACGCGCACCGGCTCCGCCCGTGGACAACCGCAAGCGGTTGCCCACCGTCGAGCGCCTTTGCCCACA
ACCCGGCGGCCGGCCGCAACAGATCGTTTTATAAATTTTTTTTTTGAAAAAGAAAAAGCCCGAAAGGCG
GCAACCTCTCGGGCTTCTGGATTTCCGATCCCCGGAATTAGATCCGTTTAAACTACGTAAGATCGATCTT
GGCAGGATATATTGTGGTGTAAACGTTCCTGCGGCGGTCGAGATGGATCTTGGCAGGATATATTGTGGTG
TAAACGTTCCT

The Cauliflower mosaic virus (CaMV) 35S promoter is marked in light red. The **TMV RNA dependent RNA polymerase (RdRp)** is writen in dark, green, bold letters. The TMV moviment protein (MP) is marked in green, and the TMV coat protein (CP) is marked in dark yellow and white letters (with the ATG codon mutated to AGA underlined) . The Hepatitis delta virus (HDV)-derived ribozyme is marked in salmon color. The CaMV 35S terminator is marked in bright red. The right border (RB) is marked in dark grey and white letters. The *E. coli* pUC replication origin is marked in light grey and dark blue letters. The Kanamycin selection marker is marked in light grey and green letters. The *A. tumefaciens* pSa replication origin is marked in light grey and bright blue letters. Finally, the duplicated left border (LB) is marked in black and white letters.

The sequence between the symbols "[" and "]" was substituted by the GoI including PeAfpA from *Penicillium expansum*, PdAfpB from *Penicillium digitatum*, GFP and the signaling peptides from *Nicotiana benthamiana* osmotin protein (AP24sp) and from *Nicotiana sylvestris* chitinases (VS).

> AP24sp-PeAfpA (246bp)

ATGTCCAACAACATGGGCAACCTCAGGTCCTCCTTCGTCTTCTTCCTCCTGGCCCTGGTGACCT
ACACTTATGCAGTTCTTTATACTGGTCAATGTTTTAAGAAGGATAATATTTGTAAGTATAAGGT
TAATGGTAAGCAAAATATTGCTAAGTGTCCTAGTGCTGCTAATAAGCGTTGTGAAAAGGATAAG
AATAAGTGTACTTTTGATAGTTATGATCGTAAGGTTACTTGTGATTTTCGTAAG

Protein product:

MSNNMGNLRSSFVFFLLALVTYTYAVLYTGQCFKKDNICKYKVNGKQNIAKCPSAANKRCEKDK
NKCTFDSYDRKVTCDFRK*

> AP24sp-PeAfpA-VS (276bp)

ATGTCCAACAACATGGGCAACCTCAGGTCCTCCTTCGTCTTCTTCCTCCTGGCCCTGGTGACCT
ACACTTATGCAGTTCTTTATACTGGTCAATGTTTTAAGAAGGATAATATTTGTAAGTATAAGGT
TAATGGTAAGCAAAATATTGCTAAGTGTCCTAGTGCTGCTAATAAGCGTTGTGAAAAGGATAAG
AATAAGTGTACTTTTGATAGTTATGATCGTAAGGTTACTTGTGATTTTCGTAAGGGCAACGGCC
TCCTGGTCGACACCATGTGA

Protein product:

MSNNMGNLRSSFVFFLLALVTYTYAVLYTGQCFKKDNICKYKVNGKQNIAKCPSAANKRCEKDK
NKCTFDSYDRKVTCDFRKGNGLLVDTM*

> AP24sp-PdAfpB (246bp)

ATGTCCAACAACATGGGCAACCTCAGGTCCTCCTTCGTCTTCTTCCTCCTGGCCCTGGTGACCT
ACACTTATGCAAGTAAATACGGAGGACAATGCAGTCTGAAACACAACACGTGCACGTACCTGAA
GGGTGGAAGAAACGTTATTGTCAACTGCGGTTCGGCTGCCAATAAGAGGTGCAAGTCTGATCGC
CACCACTGTGAATACGATGAGCACCACAGGAGGGTTGACTGCCAGACTCCAGTT

Protein product:

MSNNMGNLRSSFVFFLLALVTYTYASKYGGQCSLKHNTCTYLKGGRNVIVNCGSAANKRCKSDR
HHCEYDEHHRRVDCQTPV*

> AP24sp-PdAfpB-VS (276bp)

ATGTCCAACAACATGGGCAACCTCAGGTCCTCCTTCGTCTTCTTCCTCCTGGCCCTGGTGACCT
ACACTTATGCAAGTAAATACGGAGGACAATGCAGTCTGAAACACAACACGTGCACGTACCTGAA
GGGTGGAAGAAACGTTATTGTCAACTGCGGTTCGGCTGCCAATAAGAGGTGCAAGTCTGATCGC
CACCACTGTGAATACGATGAGCACCACAGGAGGGTTGACTGCCAGACTCCAGTTGGCAACGGCC
TCCTGGTCGACACCATGTGA

Protein product:

MSNNMGNLRSSFVFFLLALVTYTYASKYGGQCSLKHNTCTYLKGGRNVIVNCGSAANKRCKSDR
HHCEYDEHHRRVDCQTPVGNGLLVDTM*

> GFP (720bp)

ATGGTGAGCAAGGGCGAGGAGCTGTTCACCGGGGTGGTGCCCATCCTGGTCGAGCTGGACGGCG
ACGTAAACGGCCACAAGTTCAGCGTGTCCGGCGAGGGCGAGGGCGATGCCACCTACGGCAAGCT
GACCCTGAAGTTCATCTGCACCACCGGCAAGCTGCCCGTGCCCTGGCCCACCCTCGTGACCACC
CTGACCTACGGCGTGCAGTGCTTCAGCCGCTACCCCGACCACATGAAGCAGCACGACTTCTTCA
AGTCCGCCATGCCCGAAGGCTACGTCCAGGAGCGCACCATCTTCTTCAAGGACGACGGCAACTA
CAAGACCCGCGCCGAGGTGAAGTTCGAGGGCGACACCCTGGTGAACCGCATCGAGCTGAAGGGC
ATCGACTTCAAGGAGGACGGCAACATCCTGGGGCACAAGCTGGAGTACAACTACAACAGCCACA
ACGTCTATATCATGGCCGACAAGCAGAAGAACGGCATCAAGGTGAACTTCAAGATCCGCCACAA
CATCGAGGACGGCAGCGTGCAGCTCGCCGACCACTACCAGCAGAACACCCCCATCGGCGACGGC
CCCGTGCTGCTGCCCGACAACCACTACCTGAGCACCCAGTCCGCCCTGAGCAAAGACCCCAACG
AGAAGCGCGATCACATGGTCCTGCTGGAGTTCGTGACCGCCGCCGGGATCACTCTCGGCATGGA
CGAGCTGTACAAGTGA

Protein product:

MVSKGEELFTGVVPILVELDGDVNGHKFSVSGEGEGDATYGKLTLKFICTTGKLPVPWPTLVTT
LTYGVQCFSRYPDHMKQHDFFKSAMPEGYVQERTIFFKDDGNYKTRAEVKFEGDTLVNRIELKG
IDFKEDGNILGHKLEYNYNSHNVYIMADKQKNGIKVNFKIRHNIEDGSVQLADHYQQNTPIGDG
PVLLPDNHYLSTQSALSKDPNEKRDHMVLLEFVTAAGITLGMDELYK*

The apoplastic signaling peptide (AP24sp) from *Nicotiana benthamiana* osmotin protein is marked in Orange. The vacuolar signaling peptide (VS) from *Nicotiana sylvestris* is marked in bright blue. The protein PeAfpA from *Penicillium expansum* is marked in yellow. The protein PdAfpB from *Penicillium digitatum* is marked in light green. The GFP protein is marked in bright green.
