# Supplementary material for: High yield production of the antifungal proteins PeAfpA and PdAfpB by vacuole targeting in a TMV‐based expression vector
Source: Plant Biotechnol J. 2025 May 3;24(1):313–27. doi: 10.1111/pbi.70093 (PMC12854906; doi:10.1111/pbi.70093)
Supplement: Supplementary file 2 — Figure S2 Control incubations for TEM experiments. [file PBI-24-313-s003.pptx]

## Slide 1
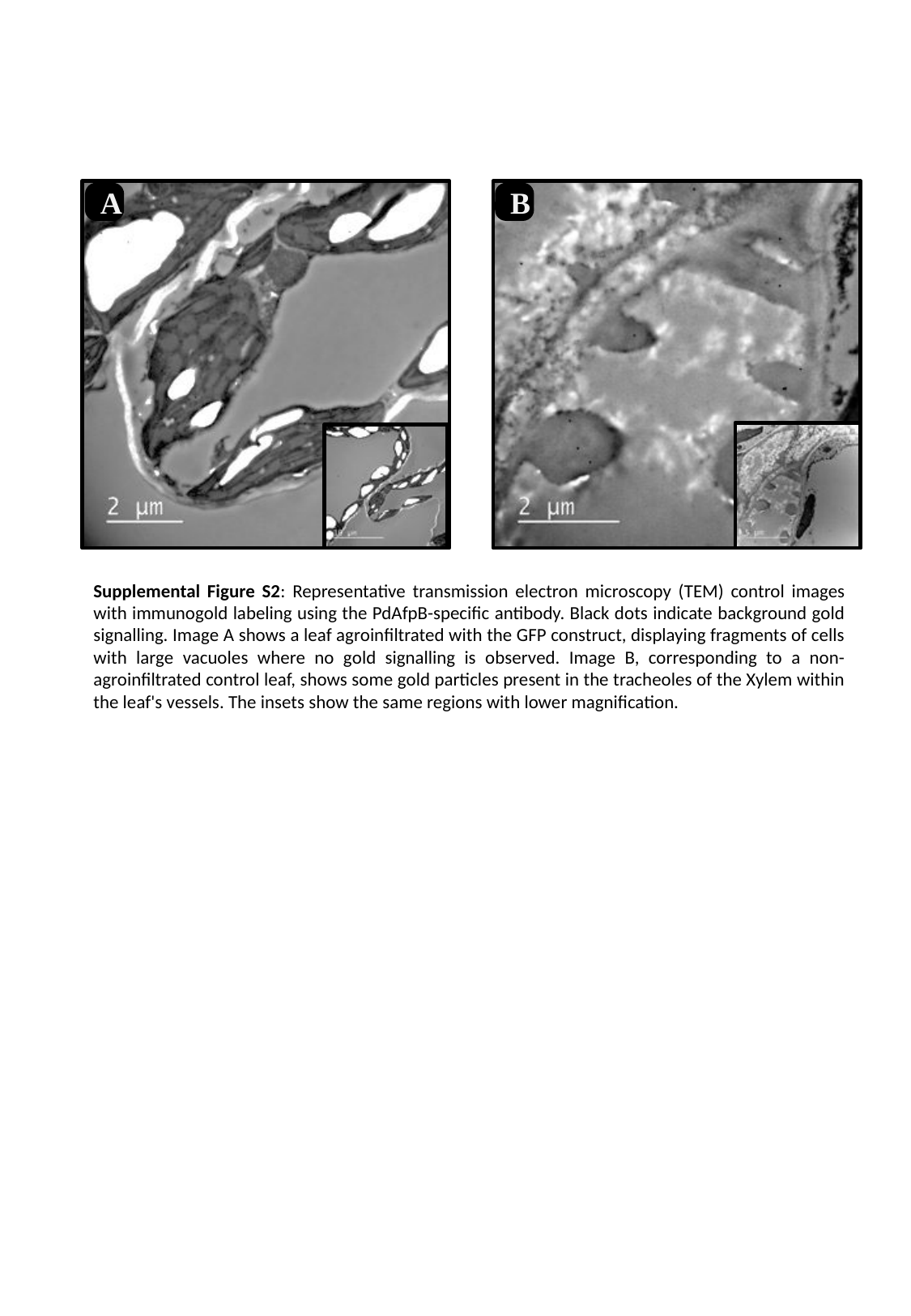

B
A
Supplemental Figure S2: Representative transmission electron microscopy (TEM) control images with immunogold labeling using the PdAfpB-specific antibody. Black dots indicate background gold signalling. Image A shows a leaf agroinfiltrated with the GFP construct, displaying fragments of cells with large vacuoles where no gold signalling is observed. Image B, corresponding to a non-agroinfiltrated control leaf, shows some gold particles present in the tracheoles of the Xylem within the leaf's vessels. The insets show the same regions with lower magnification.
