# Supplementary material for: High yield production of the antifungal proteins PeAfpA and PdAfpB by vacuole targeting in a TMV‐based expression vector
Source: Plant Biotechnol J. 2025 May 3;24(1):313–27. doi: 10.1111/pbi.70093 (PMC12854906; doi:10.1111/pbi.70093)
Supplement: Supplementary file 3 — Table S1 Mass spectrometry peptide analysis of expressed AFPs. [file PBI-24-313-s004.docx]

**Supplementary Table S1**

Identification of antifungal proteins in plants agroinfiltrated with TMV-based expression vector and detected peptides from mass spectrometry analysis of gel-separated proteins

| Protein | Score | Prot. coverage | T. cover | Unique peptides | Peptides | PSMs |
| --- | --- | --- | --- | --- | --- | --- |
| AP24-PeAfpA | 184.96 | 57.89 | 43.96 | 14 | 18 | 67 |
| AP24-PeAfpA-VS | 29.29 | 64.91 | 46.34 | 8 | 10 | 13 |
| AP24-PeAfpB | 92.78 | 70.16 | 48.78 | 13 | 18 | 35 |
| AP24-PeAfpB-VS | 86.35 | 68.42 | 48.78 | 11 | 15 | 29 |

| Protein | Sequence^1^ | PSMs | q-value | PEP |
| --- | --- | --- | --- | --- |
| AP24-PeAfpA | avlytgqcfk | 3 | 0.000 | 0.001 |
|  | ctfdsydr | 7 | 0.000 | 0.001 |
|  | ctfdsydrk | 5 | 0.001 | 0.010 |
|  | dknkctfdsydr | 3 | 0.001 | 0.011 |
|  | dknkctfdsydr | 3 | 0.000 | 0.000 |
|  | nkctfdsydr | 4 | 0.001 | 0.006 |
|  | nkctfdsydrk | 3 | 0.000 | 0.000 |
|  | TYAvlytgqcfk | 1 | 0.000 | 0.001 |
|  | vlytgqcfk | 3 | 0.007 | 0.062 |
|  | vlytgqcfk | 7 | 0.001 | 0.005 |
|  | vlytgqcfkk | 3 | 0.001 | 0.005 |
|  | vlytgqcfkk | 5 | 0.001 | 0.003 |
|  | VTYTYAvlytgqcfk | 2 | 0.011 | 0.085 |
|  | YAvlytgqcfk | 3 | 0.000 | 0.000 |
|  | YAvlytgqcfkk | 2 | 0.001 | 0.007 |
|  | ykvngkqniak | 8 | 0.001 | 0.009 |
|  | ykvngkqniak | 4 | 0.000 | 0.001 |
|  | YTYAvlytgqcfk | 1 | 0.000 | 0.001 |
| AP24-PeAfpA-VS | YAvlytgqcfk | 1 | 0.000 | 0.000 |
|  | vlytgqcfk | 1 | 0.000 | 0.033 |
|  | vlytgqcfk | 3 | 0.000 | 0.035 |
|  | vlytgqcfkk | 1 | 0.000 | 0.007 |
|  | ykvngkqniak | 1 | 0.000 | 0.001 |
|  | ctfdsydr | 2 | 0.000 | 0.024 |
|  | kvtcdfr | 1 | 0.000 | 0.004 |
|  | Avlytgqcfk | 1 | 0.007 | 0.070 |
|  | ctfdsydrk | 1 | 0.022 | 0.120 |
|  | vlytgqcfkk | 1 | 0.035 | 0.182 |

| Protein | Sequence | PSM's | q-value | PEP |
| --- | --- | --- | --- | --- |
| AP24-PeAfpB | sdrhhceydehhr | 1 | 0.000 | 0.000 |
|  | rnvivncgsaankr | 1 | 0.000 | 0.001 |
|  | nvivncgsaank | 4 | 0.000 | 0.004 |
|  | vivncgsaank | 1 | 0.000 | 0.004 |
|  | hhceydehhrr | 5 | 0.000 | 0.002 |
|  | skyggqcslk | 2 | 0.000 | 0.001 |
|  | hhceydehhr | 4 | 0.000 | 0.001 |
|  | ggrnvivncgsaankr | 1 | 0.004 | 0.063 |
|  | yggqcslk | 2 | 0.004 | 0.022 |
|  | nvivncgsaank | 4 | 0.004 | 0.022 |
|  | skyggqcslk | 1 | 0.004 | 0.029 |
|  | vivncgsaankr | 2 | 0.024 | 0.078 |
|  | nvivncgsaan | 1 | 0.026 | 0.094 |
|  | nvivncgsaankr | 1 | 0.026 | 0.104 |
|  | yggqcslk | 2 | 0.035 | 0.136 |
|  | nvivncgsaankr | 1 | 0.041 | 0.158 |
|  | sdrhhceydehhrr | 1 | 0.041 | 0.178 |
|  | nvivncgsaankr | 1 | 0.043 | 0.185 |
| AP24-PeAfpB-VS | ggrnvivncgsaankr | 1 | 0.000 | 0.223 |
|  | nvivncgsaankr | 2 | 0.000 | 0.198 |
|  | hhceydehhr | 2 | 0.000 | 0.241 |
|  | nvivncgsaank | 3 | 0.000 | 0.162 |
|  | nvivncgsaank | 4 | 0.000 | 0.247 |
|  | rnvivncgsaankr | 1 | 0.000 | 0.146 |
|  | nvivncgsaankr | 3 | 0.000 | 0.237 |
|  | sdrhhceydehhrr | 1 | 0.000 | 0.225 |
|  | nvivncgsaankr | 1 | 0.003 | 0.294 |
|  | hhceydehhrr | 4 | 0.003 | 0.269 |
|  | yggqcslk | 1 | 0.023 | 0.372 |
|  | nvivncgsaan | 1 | 0.024 | 0.400 |
|  | yggqcslk | 2 | 0.024 | 0.385 |
|  | ivncgsaankr | 1 | 0.026 | 0.460 |
|  | skyggqcslk | 2 | 0.029 | 0.466 |

^1^  Uppercase letters correspond to amino acids from signalling peptides, while lowercase letters indicate residues from the sequence of the corresponding Afp construct.
