# Supplementary material for: High yield production of the antifungal proteins PeAfpA and PdAfpB by vacuole targeting in a TMV‐based expression vector
Source: Plant Biotechnol J. 2025 May 3;24(1):313–27. doi: 10.1111/pbi.70093 (PMC12854906; doi:10.1111/pbi.70093)
Supplement: Supplementary file 4 — Table S2 Primers used in this work. [file PBI-24-313-s002.docx]

**Supplementary Table S2.** Primers used in this work

| **Primer name** | **Sequence** |
| --- | --- |
| **CP:AP24_f** | 5’-CTCAGTTCGTGTTCTTGTCAATGTCCAACAACATGGGC-3’ |
| **AfpA:CP_r** | 5’-CTACCTCAAGTTGCAGGACCTTA CTTACGAAAATCACAAGTAACC-3’ |
| **AP24_f** | 5’-CACCATGTCCAACAACATGGGCAAC-3’ |
| **AfpAVS_r** | 5’-TCACATGGTGTCGACCAGGAGGCCGTTGCCCTTACGAAAATCACAAGTAACC-3’ |
| **VS:CP_r** | 5’-CTACCTCAAGTTGCAGGACCTCACATGGTGTCGACCAG-3’ |
| **AfpBVS_r** | 5’-TCACATGGTGTCGACCAGGAGGCCGTTGCCAACTGGAGTCTGGCAGTC-3’ |
| **BcCutA_f** | 5’-AGCCTTATGTCCCTTCCCTTG-3’ |
| **BcCutA_r** | 5’-GAAGAGAAATGGAAAATGGTGAG-3’ |
| **SlActin_f** | 5’-GGTGTGATGGTGGGTATGG-3’ |
| **SlActin_r** | 5’-GCTGACAATTCCGTGCTC-3’ |
